# Structure, Properties, and *In Vitro* Behavior of Heat-Treated Calcium Sulfate Scaffolds Fabricated by 3D Printing

**DOI:** 10.1371/journal.pone.0151216

**Published:** 2016-03-21

**Authors:** Mitra Asadi-Eydivand, Mehran Solati-Hashjin, Seyedeh Sara Shafiei, Sepideh Mohammadi, Masoud Hafezi, Noor Azuan Abu Osman

**Affiliations:** 1 Department of Biomedical Engineering, Faculty of Engineering, University of Malaya, 50603, Kuala Lumpur, Malaysia; 2 Biomedical Engineering Faculty, Amirkabir University of Technology, 15914, Tehran, Iran; 3 Biomaterials Center of Excellence, Amirkabir University of Technology, 15914, Tehran, Iran; 4 Stem Cell and Regenerative Medicine Group, National Institute of Genetic Engineering and Biotechnology, Tehran, 14965/161, Iran; University Hospital of Modena and Reggio Emilia, ITALY

## Abstract

The ability of inkjet-based 3D printing (3DP) to fabricate biocompatible ceramics has made it one of the most favorable techniques to generate bone tissue engineering (BTE) scaffolds. Calcium sulfates exhibit various beneficial characteristics, and they can be used as a promising biomaterial in BTE. However, low mechanical performance caused by the brittle character of ceramic materials is the main weakness of 3DP calcium sulfate scaffolds. Moreover, the presence of certain organic matters in the starting powder and binder solution causes products to have high toxicity levels. A post-processing treatment is usually employed to improve the physical, chemical, and biological behaviors of the printed scaffolds. In this study, the effects of heat treatment on the structural, mechanical, and physical characteristics of 3DP calcium sulfate prototypes were investigated. Different microscopy and spectroscopy methods were employed to characterize the printed prototypes. The *in vitro* cytotoxicity of the specimens was also evaluated before and after heat treatment. Results showed that the as-printed scaffolds and specimens heat treated at 300°C exhibited severe toxicity *in vitro* but had almost adequate strength. By contrast, the specimens heat treated in the 500°C–1000°C temperature range, although non-toxic, had insufficient mechanical strength, which was mainly attributed to the exit of the organic binder before 500°C and the absence of sufficient densification below 1000°C. The sintering process was accelerated at temperatures higher than 1000°C, resulting in higher compressive strength and less cytotoxicity. An anhydrous form of calcium sulfate was the only crystalline phase existing in the samples heated at 500°C–1150°C. The formation of calcium oxide caused by partial decomposition of calcium sulfate was observed in the specimens heat treated at temperatures higher than 1200°C. Although considerable improvements in cell viability of heat-treated scaffolds were observed in this study, the mechanical properties were not significantly improved, requiring further investigations. However, the findings of this study give a better insight into the complex nature of the problem in the fabrication of synthetic bone grafts and scaffolds via post-fabrication treatment of 3DP calcium sulfate prototypes.

## Introduction

The rapidly growing discipline of tissue engineering is one of the most hopeful approaches for developing engineered substitutes for damaged bone [1]. Scaffolds for bone tissue engineering (BTE) applications are anticipated to have certain properties to encourage bone regeneration. Scaffolds are highly porous structures with interconnected pores. They should ideally be biocompatible, mechanically reliable, biodegradable, osteoconductive, and biomimetic [2–6].

Many experts believe that the progress of BTE is seemingly associated with the improvements in scaffold technology [7, 8]. Numerous multidisciplinary studies have been carried out in this field, from design and modeling to material processing and post-treatments, as well as *in vitro* and *in vivo* biological evaluations [5, 9–11]. Various processing techniques, such as salt leaching [12], foam replica [13], gas foaming [14], freeze casting [15], and electrospinning [16], have been used to fabricate scaffolds. However, most of these methods cannot fully control the structural properties and reproducibility of the scaffolds.

Therefore, a great deal of attention to additive manufacturing methods has been raised in recent years. These methods are a group of advanced fabrication methods, generally branded as solid freeform fabrication (SFF), in which 3D articles can be constructed layer by layer in an additive manner straight from data obtained by computer-aided design (CAD), computed tomography, and magnetic resonance imaging. Rapid prototyping techniques show the ability for the fabrication of predefined, customized, and reproducible scaffolds with tailored architecture and porosity [11, 17–20].

Among the SFF methods, powder-based 3D printing (3DP) has been widely used to construct BTE scaffolds. In the 3DP method, the geometry, shape, and internal porous structure of the implant are first designed in a CAD environment. Afterward, the CAD model is transformed into image slices. The scaffold is then printed in a layer-by-layer manner by repetitive stacking of powder layers. Binder droplets are selectively jetted to the pre-deposited thin layer of the powder to fabricate a model based on a sequence of mathematically sliced cross sections of the CAD file. This method is a promising approach in the field of tissue engineering, specifically for bone substitute fabrication [21–24]. A large number of biocompatible ceramic and composite materials can be processed using 3DP [4, 18, 25].

Calcium sulfate was introduced as a bone substitute material in 1892 by Dreesman [26]. In 1961, Peltier introduced calcium sulfate as a suitable material for filling bone defects [26]. Since then, further studies have been conducted on calcium sulfate [27–29]. Moreover, the composites of calcium sulfate have been manufactured under commercial brands [29, 30] for BTE applications. Calcium sulfate is biocompatible, osteoconductive, and highly resorbable [31–35].

Previous reports [36, 37] suggested that the release of calcium ions from calcium sulfate implants as a result of the dissolution process increases the number of osteoblasts and osteoclasts at the wound site by enhancing cellular genesis, thereby enhancing bone regeneration. Calcium sulfate can also be considered a promising vehicle for the delivery of therapeutic compounds, such as drugs, antibiotics, proteins, and platelet-derived growth factors [38, 39]. Therefore, calcium sulfates show several useful characteristics as an ideal bone tissue regenerative biomaterial. With recent advances in ceramic science and engineering, calcium sulfates can be considered suitable materials for BTE applications [40, 41].

Three common forms of available calcium sulfates are dihydrate or gypsum (CaSO_4_.2H_2_O), hemihydrate or basanite (CaSO_4_.0.5H_2_O), and anhydrous calcium sulfate or anhydrite (CaSO_4_). When medical grade calcium sulfate, a highly degradable biocompatible material, is implanted inside the body, the by-products of the degradation process do not cause adverse effects in the body [34, 42]. Calcium sulfate hemihydrate as hydraulic cement is one of the most widely used ceramics in printing 3D objects. The water-based binder reacts with the powder particles, resulting in the formation of calcium sulfate dihydrate crystals [18, 21].

As the compressive strength are 5–10 MPa and young’s modulus are 50–100 MPa for cancellous bones [43–45], so the major weakness of 3DP porous bioceramics is their relatively low mechanical performance because of the brittle nature of ceramic materials. Therefore, a post-processing treatment is usually employed to improve the strength of the printed objects. Recent studies on post-processing of 3DP scaffolds for tissue engineering applications have disclosed that post treatments may significantly influence the physical and chemical properties of the fabricated 3D objects, as well as their *in vitro* degradation properties [27, 46]. The two most common post-processing procedures are sintering and infiltration [46–50].

Previous studies on 3DP of ceramic porous structures have shown remarkable increases in compression strength of calcium phosphate-printed samples after sintering [17, 51–53]. Only a few studies on sintering calcium sulfate 3DP prototypes have been reported. Zhou and colleagues studied the sintering of 3DP calcium sulfate specimens up to 861°C [27]; no study on higher temperatures has been reported to date.

The present study aimed to investigate the influence of heat treatment on the mechanical, structural, physical, and *in vitro* properties of 3DP calcium sulfate prototypes. In our previous reports [54–56], we described the effect of layer thickness, layer printing delay time, dimensional accuracy, and printing orientation on the structural features and mechanical properties of calcium sulfate prototypes fabricated by the 3DP method. We used the optimal printing settings obtained from our earlier studies to fabricate the scaffolds that were heat treated in the present work. This approach attempts to combine the advantages of the 3DP process with well-established practices, such as heat treatment and sintering. We believe that the present study contributes to a better insight into the complex nature of the whole process.

## Experimental Procedure

### Materials

A commercially available calcium sulfate hemihydrate powder (zp150) was used as the printing starting material. Wetting calcium sulfate hemihydrate (CaSO_4_ +0.5H_2_O) activate the self-hydration process and subsequently, gypsum paste (CaSO4·1/2H2O+1 1/2H2O = CaSO4·2H2O) forms.

The binder was an aqueous commercial solution (zb63) containing 2-pyrrolidinone with the viscosity similar to the water. Both the powder and binder were supplied by 3D Systems Inc. (USA) and used without further treatment.

### Particle size and surface area analyses

The particle size distribution of the supplied zp150 powder was analyzed using a laser particle size analyzer (Mastersizer MV, Malvern Instruments, UK), and the *d10*, *d50*, and *d90* values were reported accordingly. A surface area analyzer (ASAP2020, Micromeritics, USA) was used to obtain the BET specific surface area value of the powder.

### Scaffold design and printing

**Fig 1:**
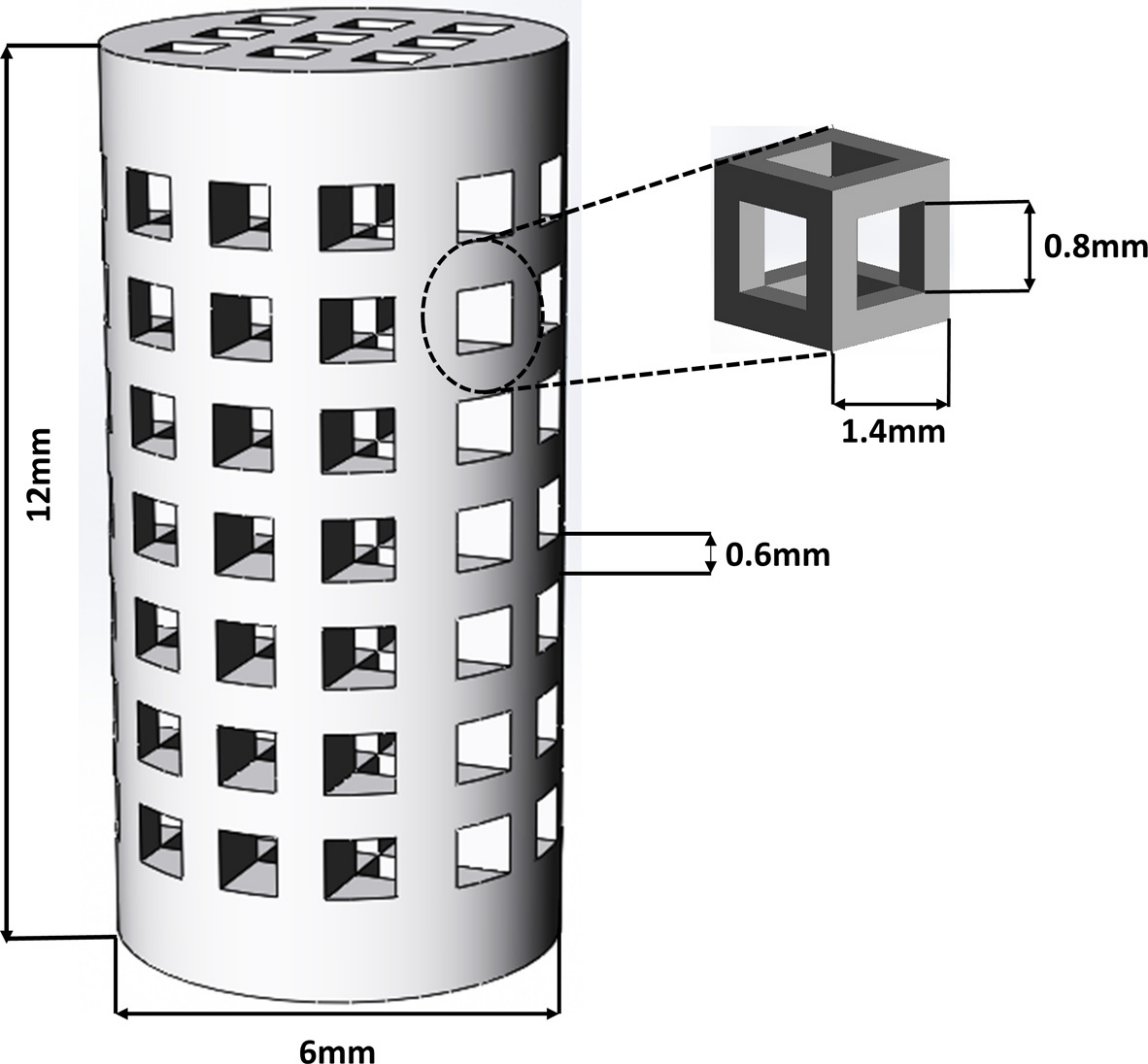
Structural details of the porous scaffold CAD and the unit cell design.

The geometry of the scaffold was chosen because it represented the typical architecture and features of a BTE scaffold considered to repair an anatomical deficiency in a biological environment [57]. Although the description of a suitable pore size is still a matter of debate [58], it is commonly agreed to be in the 100–800 μm range [59]. Porous cylinders, 12 mm in height and 6 mm in diameter, with 45.04% porosity, were designed (Fig 1) using CAD software (SolidWorks 2012) and exported as.STL file to the printer. We used the same approach from our previous work to design the scaffold prototypes for the present work [54]. In brief, the CAD files in STL format were imported to a commercial ZPrinter 450 3DP machine (3D Systems Inc., USA) to print the designed cylindrical scaffold prototypes. A layer thickness of 0.089 mm was used to slice the designed 3D structure into 2D layers; thus, the thickness of powder in each layer was the same.

**Fig 2:**
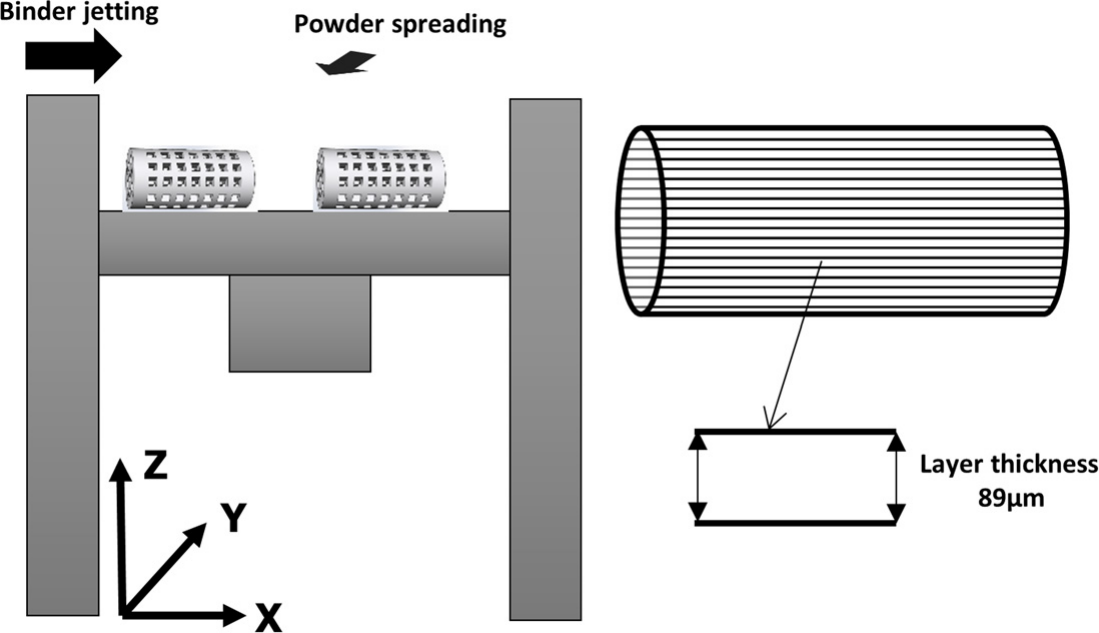
Schematic of the build bed printing layout and printing parameters.

The printing process started by filling the feeder with calcium sulfate powder. The roller spread a layer of powder from the feeder to the build area (Fig 2). A 300 ms delay time between spreading each layer was considered. The print head jetted the binder along the x-direction of the build bed. After 3DP, all the specimens were held in the machine (1.5 h, 35°C) to enhance the powder–binder setting reaction and allow the scaffolds to harden. The printed scaffold prototypes were cleaned using a soft brush and then depowdered by blowing compressed air to remove the trapped and unbound powder.

### Thermal analysis

The thermal behavior of the as-printed samples was examined by a simultaneous thermal analysis instrument (Setaram, France) in the 25°C–1300°C temperature range. Thermogravimetric analysis (TG/DTG) was performed using a thermal analysis apparatus (Mettler-Toledo, ThermoStar^TM^, Switzerland) in the 25°C–1000°C temperature range. A heating rate of 10°C/min in air was used in both experiments.

### Heat treatment

Heat treatment of the printed calcium sulfate scaffolds was carried out in an electric box furnace (XY-1600A, Nanyang Xinyu Furnaces LTD., China) equipped with a heating rate controller device. The samples were placed on an alumina foam plate and heated in air at 300°C, 500°C, 900°C, 1000°C, 1150°C, 1200°C, 1250°C, and 1300°C at a heating rate of 10°C/min. These temperatures were chosen to correspond to the thermal behavior of the printed sample observed in DTA-TG and TG-DTG thermal analyses. The cylindrical printed specimens were positioned in the furnace to stand on their base. All samples were soaked at the target temperature for 1 h, followed by furnace cooling to room temperature. The effect of heating temperature on the 3DP calcium sulfate scaffolds was then investigated.

### Composition and microstructure analysis

#### X-ray diffraction (XRD)

XRD characterization was performed in a scan angle (2θ) range of 10 to 80 using Cu-K_α_ radiation (1.54056 Å), 40 kV, 30 mA, and 0.02°s^−1^ step scan within a DY1032 diffractometer (PANalytical, The Netherlands). CrystalDiffract v1.4.7 software was used to acquire the XRD patterns. The major phases of the samples were identified using the JCPDS files.

#### Fourier transform infrared (FTIR) spectroscopy

The presence of chemical groups in the calcium sulfate powder and printed scaffolds were recognized using an FTIR spectrometer (IFS66v/S, Bruker, Germany). The spectra were collected in transmittance mode in the 4000–450 cm^−1^ range.

#### Scanning electron microscopy (SEM)

The microstructure of the printed and heat-treated specimens was assessed using SEM (Phenom Pro X, The Netherlands) equipped with energy dispersive spectroscopy (EDS). Carbon tape was used to fix the specimens on the sample holder to discharge the negative charge in the microscope. Subsequently, backscattered electron images were obtained, and the spectra of energy dispersive X-ray microanalysis were obtained.

### Mechanical testing

Uniaxial compression tests were performed on a universal testing instrument (Table top 5569, Instron, USA) equipped with a 100 kN load cell and a cross-head loading rate of 0.05 mm min^−1^. Each measurement was conducted on three identical specimens at room temperature. The maximum compressive stress registered in the stress–strain plot and the slope of the linear region before the yield point were considered compressive strength and compressive elastic modulus, respectively.

### Shrinkage and density measurement

**Fig 3:**
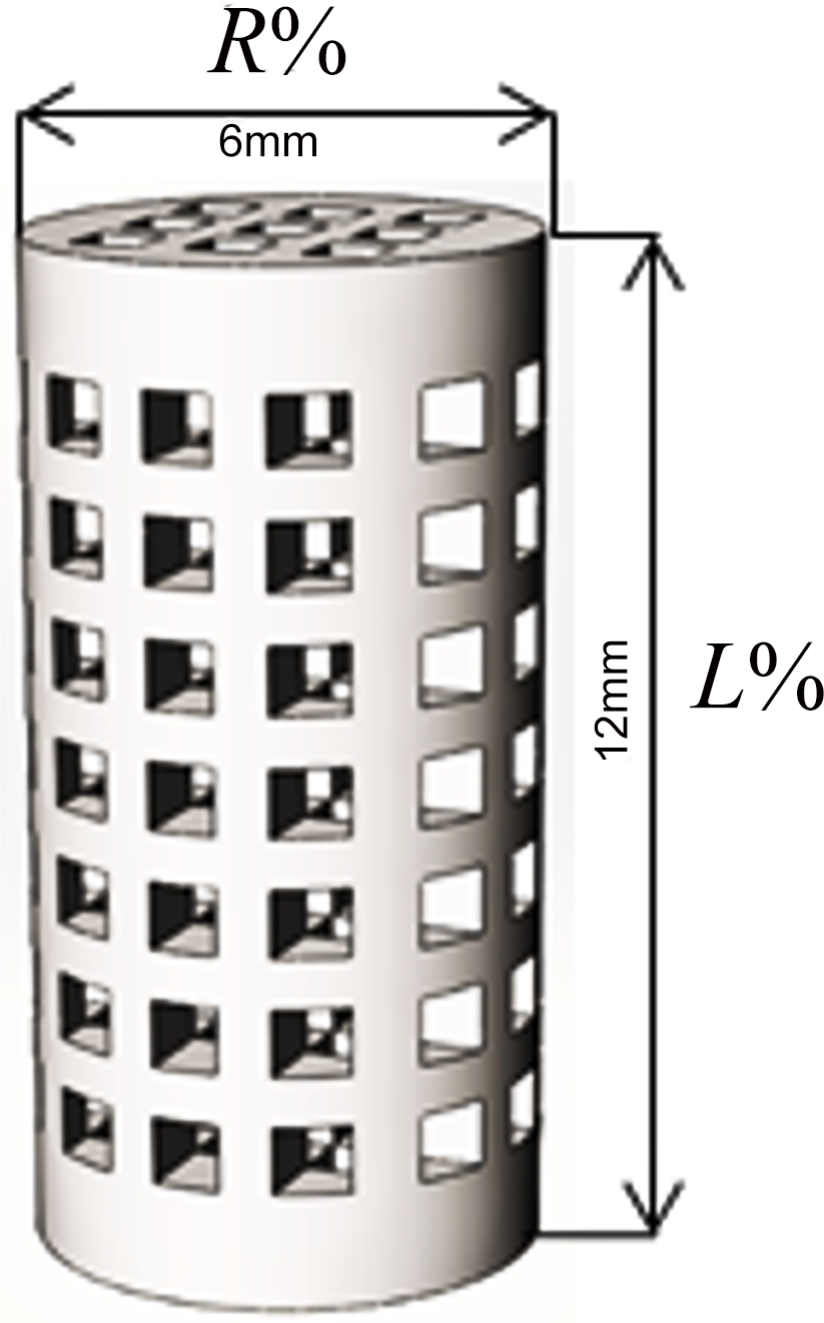
Heat treatment of the 3DP specimens results in shrinkage in both longitudinal and radial directions.

Dimensions of the test specimens were measured before and after heat treatment using a digital caliper (Mitutoyo, Model CD-6″CS) with 0–150 mm measurement range and 0.01 mm accuracy. Each feature was measured 10 times, and the average was reported for 10 samples. The percentage of longitudinal (*L*%) and radial (*R*%) shrinkage (Fig 3) was calculated according to the following equations:

$$R\%=\left[\frac{d_{H}-d_{p}}{d_{p}}\right]\times 100$$ (1)

$$L\%=\left[\frac{l_{H}-l_{P}}{l_{P}}\right]\times 100$$ (2)

where (*d*_*H*_,*l*_*H*_) and (*d*_*P*_,*l*_*P*_) are the diameter and height of the specimens after and before heat treatment, respectively. The readings were recorded, averaged, and compared with the CAD-designed dimensions by calculating the deviation as a percentage difference.

The weight of the samples was measured by a semi-micro analytical balance (GR-200, A&D, Japan) to four decimal points. The bulk density of the printed samples was calculated using the mass and bulk volume data obtained by weighing and measuring, respectively.

### Cytotoxicity studies

MG63 (human osteoblast-like osteosarcoma) cells were obtained from the National Cell Bank of Iran, Pasteur Institute (Tehran, Iran). Cell cultures were maintained in DMEM (L-glutamine) supplemented with 10% fetal bovine serum (FBS, Gibco) and 1% penicillin/streptomycin at 37°C in a humidified atmosphere with 5% CO_2_. Prior to cell seeding, the samples were sterilized by immersing in 70% ethanol for 1 h, followed by washing several times with sterilized PBS. Cytotoxicity assay was performed according to the ISO 1993–5 protocol. Typically, 0.1 g of powder was incubated in 1 mL of sterilized culture medium. The media were extracted for use in cellular assays at the predefined time intervals (1, 3, and 7 days). The culture medium was kept under similar conditions as a negative control. The cytotoxicity of the extracts was assessed using MTT (3-(4,5-dimethylthiazol-2-yl)-2,5-iphenyltetrazolium bromide) assay. In brief, the cells were cultured in a 24-well plate at a density of 1×10^4^ cells/well. After 24 h, the culture medium was replaced with 100 μL of extracts supplemented with 10 μL FBS. The medium was discarded after 24 h of incubation, and 100 μL of MTT solution (0.5 mg/mL in PBS) was added to each well. Following the incubation of cells for 4 h at 37°C, the dark blue formazan crystals were dissolved by adding 100 μL of DMSO per well. Finally, 100 µL of each sample was transferred to a 96-well ELISA plate, and the absorbance was measured at 570 nm. The tests were repeated for three specimens in all samples, and the cell viability percent was reported as the average absorbance of each extract group divided by that of the control group.

### Statistical analysis

Data collected from height, diameter, and weight measurements were evaluated for statistical significance using one-way ANOVA. A value of *P* < 0.001 was considered significantly different. Tests were conducted using SPSS 13.0 software (SPSS, USA).

## Results and Discussion

Among the many different rapid prototyping techniques, 3D powder printing is particularly attractive because of its rapid and inexpensive ability to form accurate structures. In our previous study [54], we avoided any further post-treatment to focus only on the design of experiment factors that affect the dimensional accuracy and mechanical behavior of the printed parts. In the present work, we subjected the 3DP scaffolds to a heating process to evaluate the influence of heat treatment on the structure, mechanical performance, and *in vitro* response of 3DP scaffolds.

### Scaffold design, printing, and structural features

**Fig 4:**
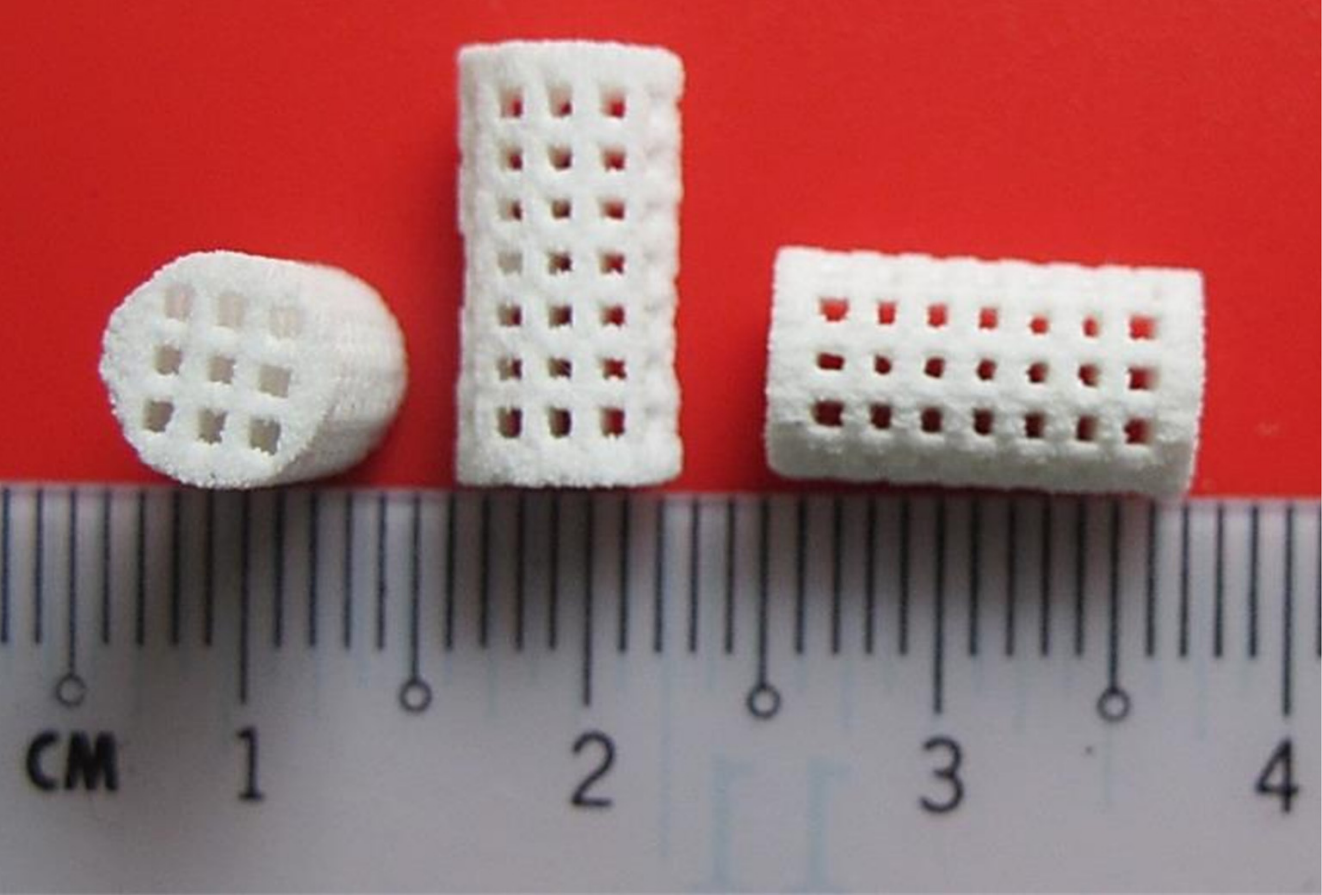
Top and side views of the fabricated scaffold using a unit cell with a 0.8 mm pore size.

The 3DP scaffold prototypes were fabricated based on 800 μm pore size and 600 μm strut size design (Fig 4). The printhead provided droplets based on an inkjet printing technique. The printhead moved very close to the powder to decrease imperfections related to jetting of the binder spray. After printing, the scaffolds possessed sufficient green strength to withstand the air-gun pressure during removal of the unbound powder.

The correlation between optimum scaffold pore size and cell activity has always been a conflicting issue in the literature [60]. Large pores (>0.5 mm) favor fast vascularization but decreases the specific surface area, which in turn limits cell attachment [61]. This phenomenon represents a potential limitation of 3DP technology because of the difficulty in removing unbound powder from the small cavities within the scaffold after the printing process.

According to Vorndran *et al*. [62], the powders must meet the following two conditions for successful application in 3DP: 1) capability to form relatively smooth and even powder layers in the thickness range of 100–200 μm, and 2) react with the binder solution during the printing process and harden consequently. The first condition is mainly related to the particle size and particle size distribution of the starting powder. A previous study [63] explained that appropriate particle sizes should be in the 20–50 μm range. Powders with relatively small particle size can be easily removed from the printed parts. However, the presence of fine particles smaller than 5 μm in the powder encourages the formation of agglomerates up to 1–2 mm in diameter, leading to a heterogeneous powder bed with sizeable grooves that make precise printing almost unachievable [21, 62].

**Fig 5:**
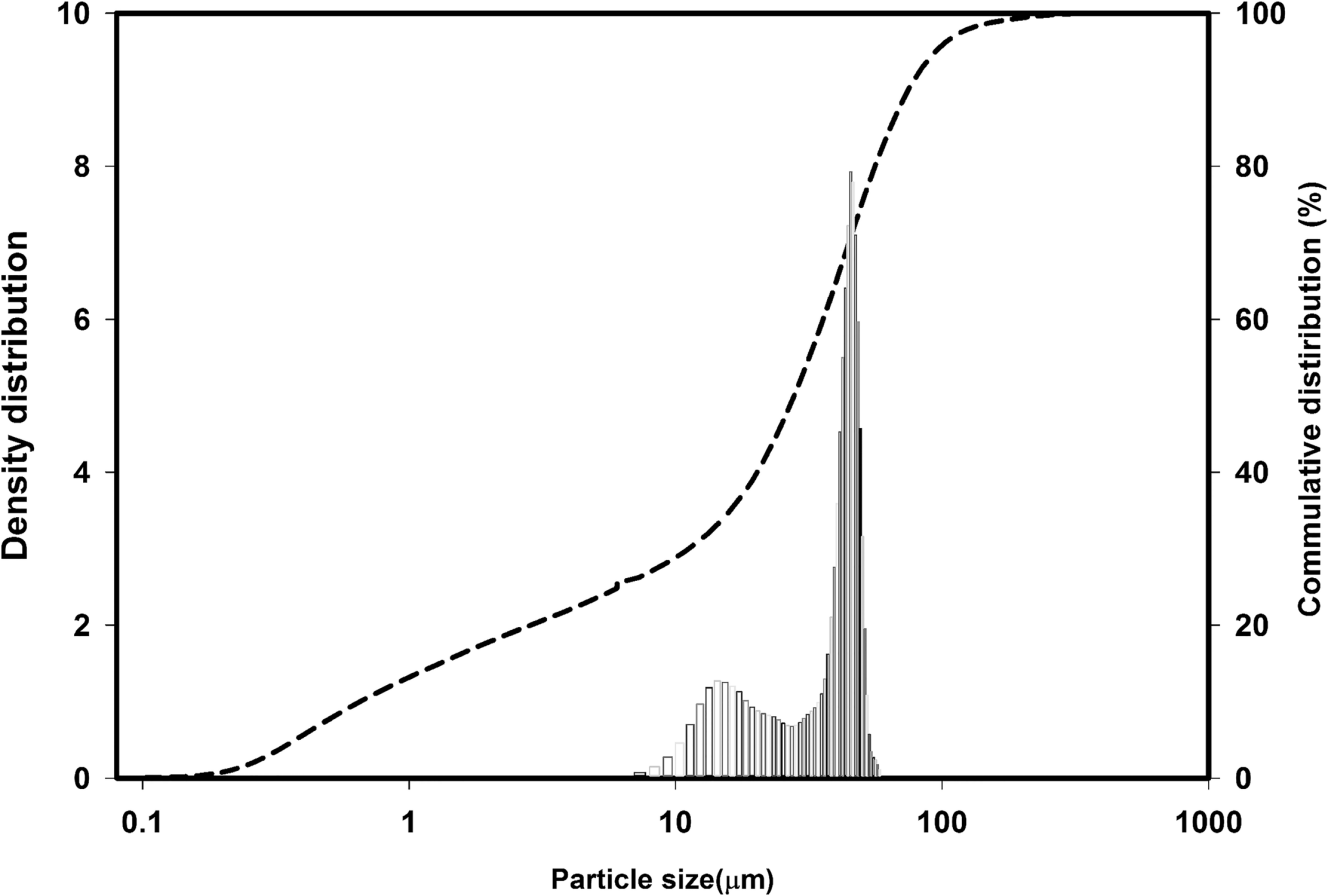
Differential and cumulative particle size distributions of the zp150 calcium sulfate starting powder.

Differential and cumulative particle size distributions of zp150 powder are shown in Fig 5. Employing a starting powder with an appropriate particle size (d_10_ = 0.64 μm, d_50_ = 27.36 μm, d_90_ = 68.83 μm) can significantly reduce the formation of unwanted agglomerates. However, this particle size range may impose a limit on the minimum feature size, particularly when printing scaffolds with complicated geometry and structure.

Powder particle size also has an influence on the layer thickness that can be attained. Thin powder layers are preferable because a relatively higher level resolution can be achieved. Meanwhile, the layer thickness must be larger than the largest particle size of the powder. Considering all the necessary factors, 89 μm was chosen as the layer thickness in the present work because the powder particles being used had a d_90_ = 68.83 μm. This powder was suitable for the printing process and enabled high printing accuracy, as well as sufficient mechanical strength, to remove the printed samples from the powder bed.

The commercially available 3DP powder consisted mainly of calcium sulfate hemihydrate and small quantities of water-soluble organic additives. The organic portion contributed in binding the powder particles in the course of the printing process to provide the necessary green strength to the printed parts.

**Fig 6:**
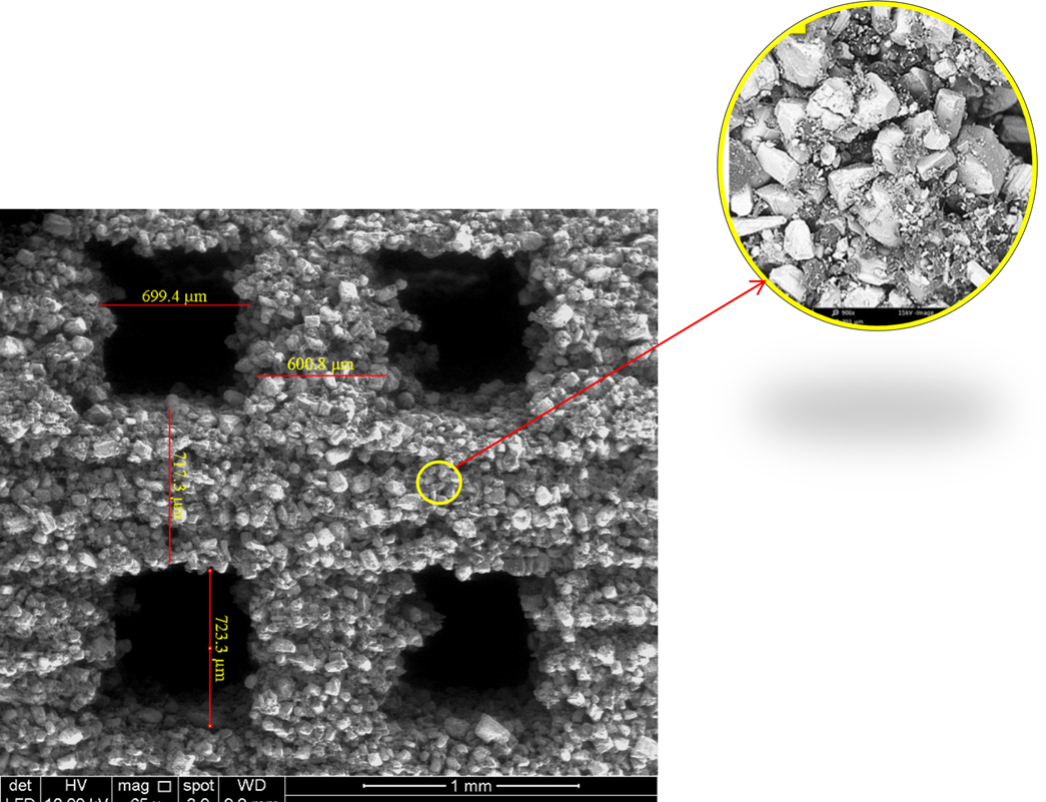
SEM image of the microstructure of the 3DP scaffold using a unit cell design with 0.8 mm pore size. The printed pore size is in the 700–750 μm range.

The microstructure of the 3DP scaffold using a unit cell design of 0.8 mm pore size is shown in Fig 6. The printed pore size was in the 700–750 μm range, and pore channels were free of loose powder, possessing rough edges.

**Fig 7:**
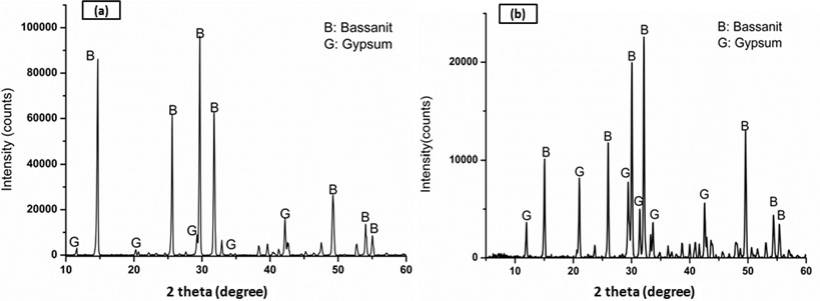
XRD patterns of a) the starting powder and b) as-printed scaffold.

Fig 7 displays the XRD spectra of the zp150 powder and as-printed sample. The XRD patterns indicated that the starting powder mainly consisted of the calcium sulfate hemihydrate (CaSO_4_.0.5H_2_O) phase. The presence of a trace amount of calcium sulfate dihydrate (CaSO_4_.2H_2_O) phase in the starting powder was most likely caused by the ability of the zp150 powder (BET specific surface area, 1.94 m^2^/g) to absorb moisture from the environment, followed by a partial hydration reaction.

**Fig 8:**
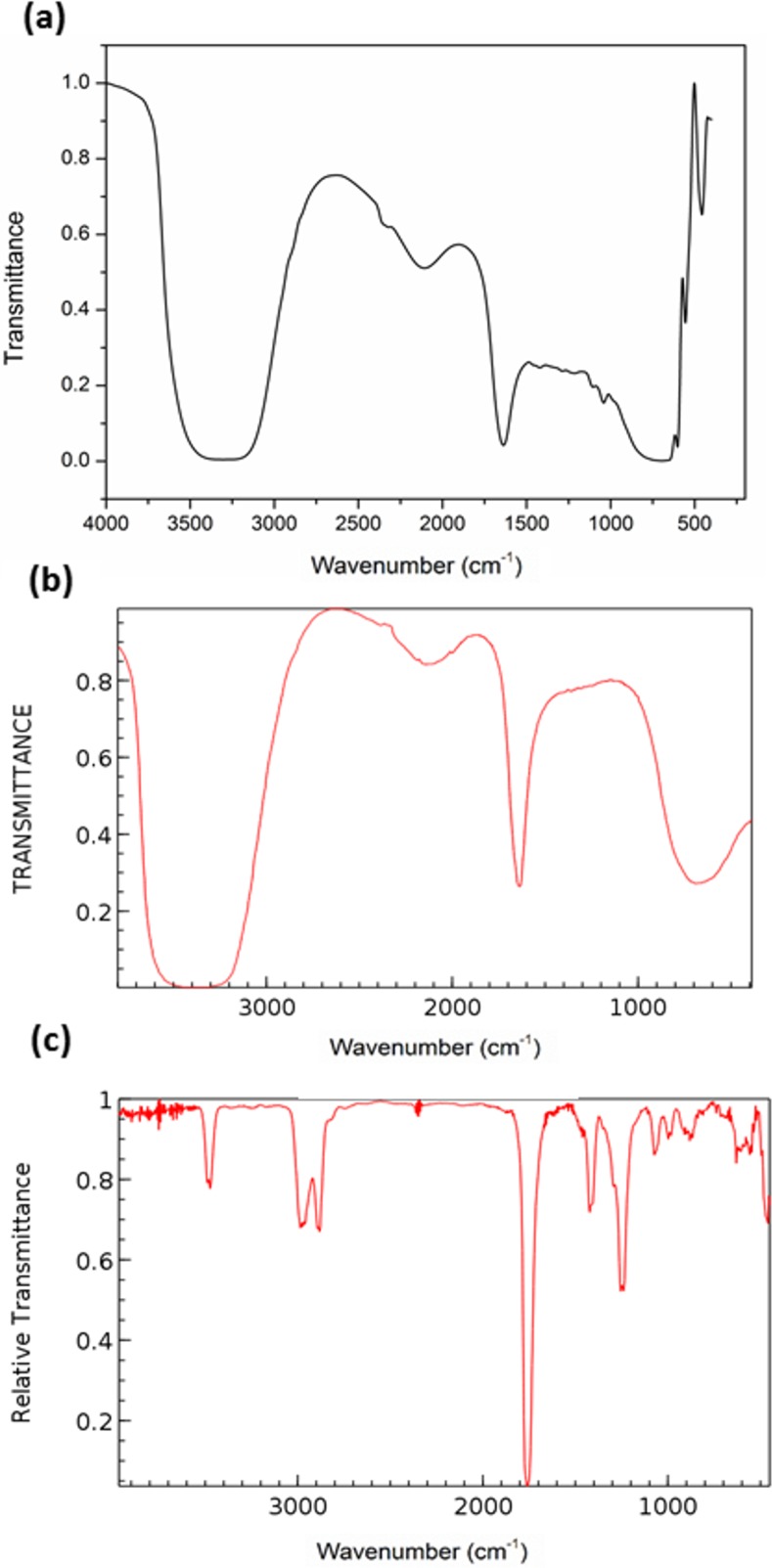
FTIR spectra of the a) zb63 binder, b) pure water [67], and c) 2-pyrrolidinone[68]

The binder used in this work was a commercial solvent containing 2-pyrrolidinone (Safety Data Sheet of zb63 binder, 3D Systems Inc., 2012). The FTIR spectra of the zb63 binder, pure water, and 2-pyrrolidinone are shown in Fig 8. A comparison of the FTIR spectra of the zb63 binder and pure water showed that the binder mostly contained water. Some of the unidentified peaks in the 700–450 cm^−1^ region of the FTIR spectrum were related to the organic components (2-pyrrolidinone) of the binder solution.

A hardening process occurred as a consequence of the hydraulic reaction between the calcium sulfate hemihydrate powder and water-based binder. The powder–binder reaction during the printing process resulted in the formation of a small amount of calcium sulfate dihydrate, along with the hemihydrate phase. The XRD spectra showed a mixture of both calcium sulfate dihydrate and hemihydrate phases in the printed scaffolds (Fig 7B). This result was probably caused by the inadequate contact between the binder and powder in the course of 3DP, and it is a sign of an incomplete chemical re action.

The phase percentage of CaSO_4_.2H_2_O and CaSO_4_.0.5H_2_O in the as-printed scaffolds was quantified using the relative intensity ratio of the corresponding and the following equation [64]:

$$\text{Percent of the phase}=\frac{\text{Intensity of major peak of the phase to be determined}}{\sum \text{Intensity of major peaks of all the phases}}\times 100$$ (3)

XRD analysis of the porous specimens revealed a higher CaSO_4_.0.5H_2_O phase quantity compared with the lesser amounts for the solid specimens. This finding indicated that the hemihydrate phase remained the major phase during the printing process, whereas the dihydrate phase was not significantly developed as a result of the powder–binder reaction. The percentages of the calcium sulfate phases in the starting powder, porous, and solid printed scaffolds are shown in Table 1.

**Table 1:** Calcium sulfate phases present in the porous and solid 3DP scaffolds calculated based on the XRD results

	CaSO_4_.0.5H_2_O (%)	CaSO_4_.2H_2_O (%)
**Powder**	94.0	6.0
**Porous**	89.0	11.0
**Solid**	63.4	36.6

**Fig 9:**
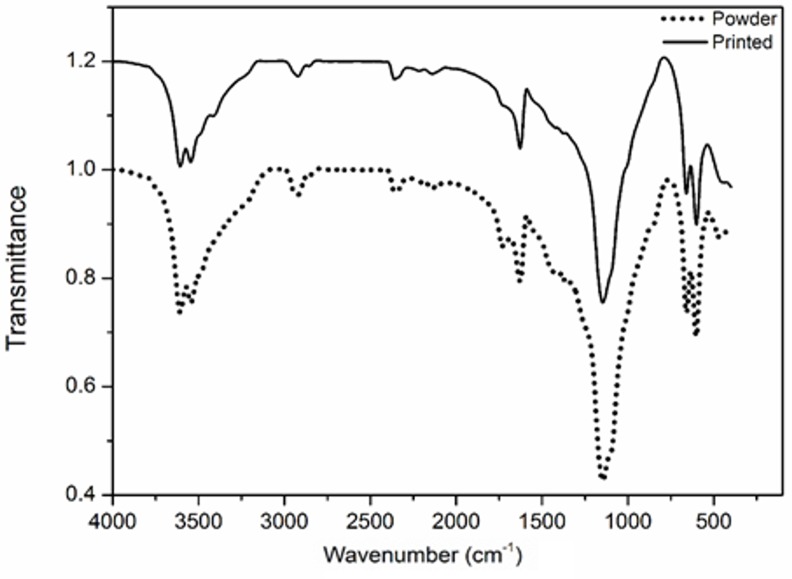
FTIR spectra of the as-printed scaffold (solid line) and ZP150 starting calcium sulfate powder (dotted line).

The FTIR spectra of the starting zp150 calcium sulfate powder and the as-printed scaffold are shown in Fig 9. The bands related to the stretching vibration of the O-H groups were detected in the 3700–3500 cm^−1^ range. The bending vibrations of the O-H bond in the H_2_O molecule were associated with the bands at 1724 and 1627 cm^−1^. The characteristic bands assigned to the vibrations of the S-O bonds in SO_4_^2−^ appeared at wave numbers 1146 cm^−1^ (stretching) and 659 and 601 cm^–1^ (bending). In the spectrum of the calcium sulfate powder (dotted line), the trace of the C-O band at 1425 and 1370 cm^−1^, as well as a weak shoulder at 900 cm^−1^, was attributed to the presence of CO_3_^2−^ groups because of the CO_2_ adsorption capacity of the relatively high surface area of the zp150 powder. This trace was less noticeable in the spectrum of the printed scaffold (solid line). The FTIR spectrum of the starting powder was very similar to that of the printed scaffold. Therefore, no significant structural changes occurred in the hydration state of the starting calcium sulfate during the 3DP process, which was in agreement with the results from XRD analysis discussed earlier.

### Effects of heat treatment on composition and microstructure

**Fig 10:**
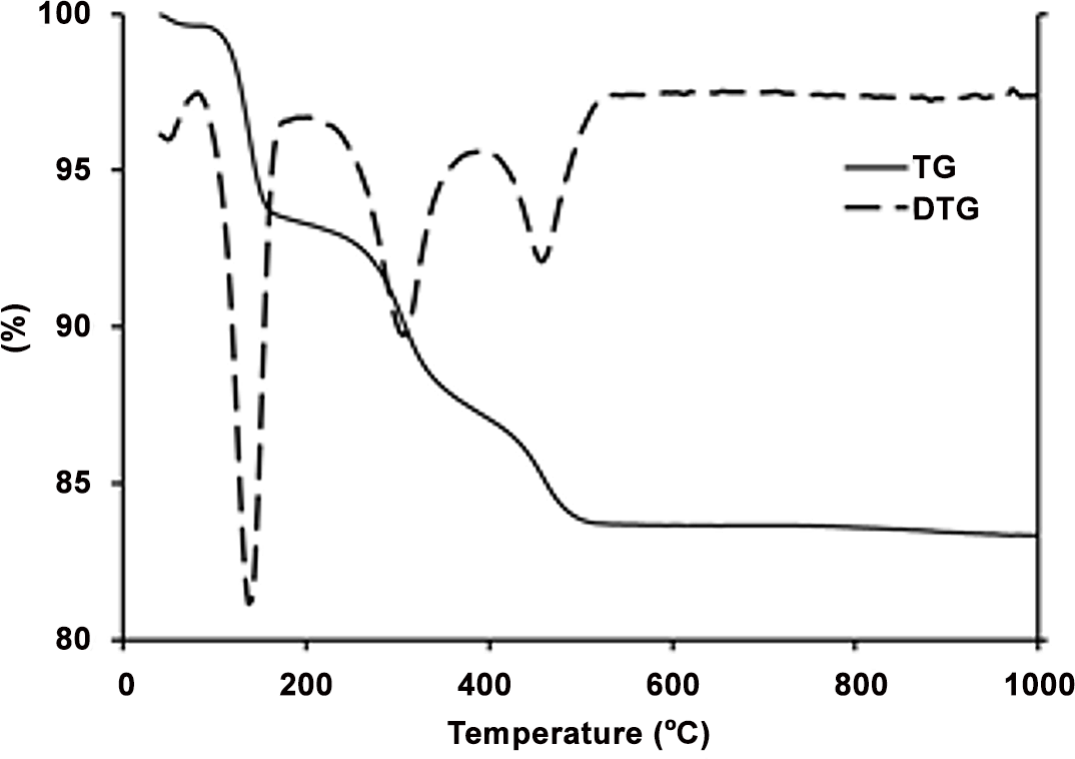
TG curve of the as-printed scaffold and its derivative (DTG).

During the heat treatment of the 3DP calcium sulfate samples, several processes occurred, including water exit, decomposition and combustion of the organic binder, and formation of new phases. Fig 10 shows the thermal behavior of the as-printed sample in the temperature range of 50°C–1000°C.

**Fig 11:**
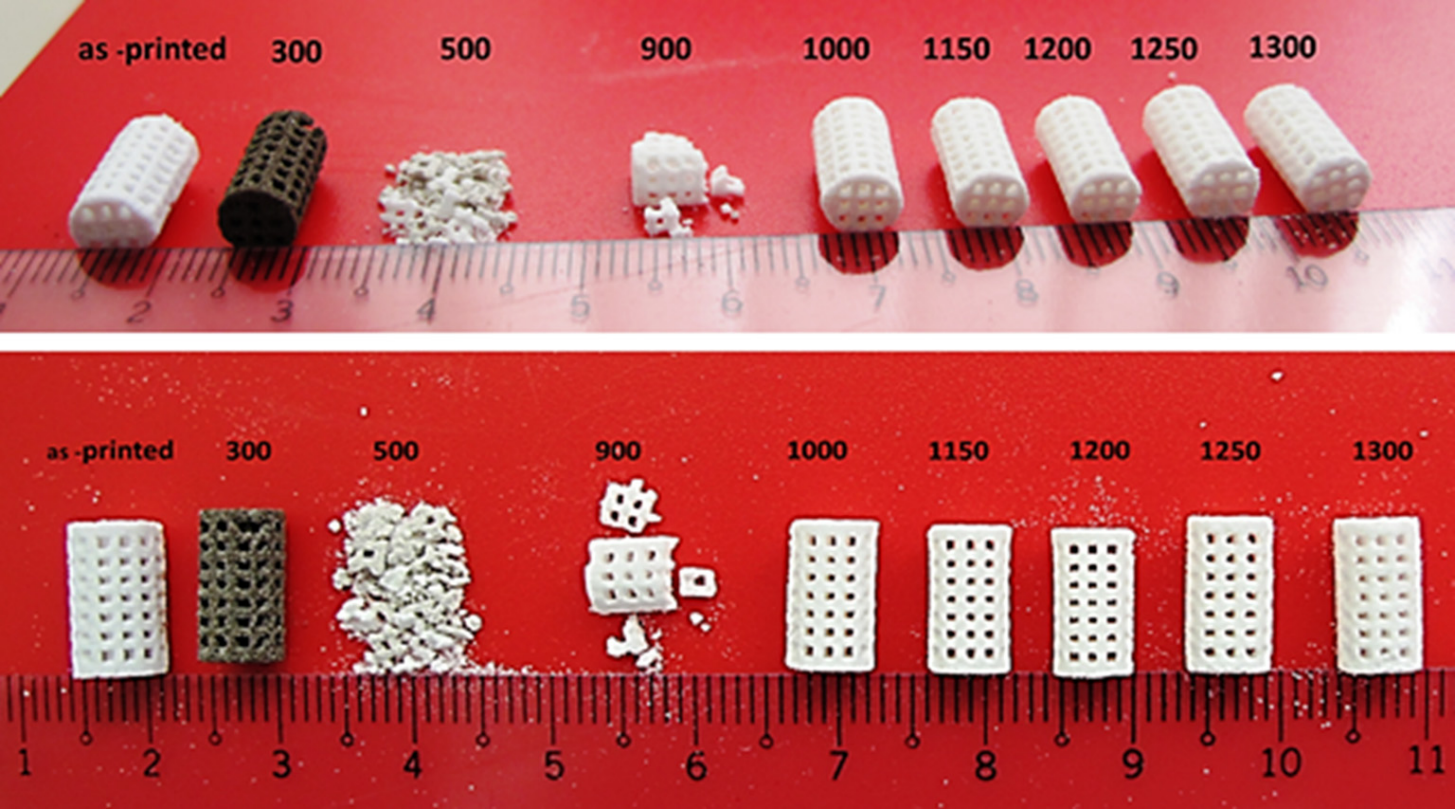
Samples heat treated at various temperatures.

The TG-DTG curves revealed a total mass loss of 14% from room temperature to 1000°C in four distinguished weight loss steps:

1. The 6.3% weight loss from room temperature to 200°C could be attributed to the loss of residual water from the sample, as well as the partial dehydration of the calcium sulfate hemihydrate phase.
2. The 6.1% weight loss between 200°C and 390°C may be mainly assigned to the burning and exit of organic species of the starting powder and binder and the completion of hemihydrate (CaSO_4_.0.5H_2_O) dehydration. In addition, the 300°C samples underwent an emphasized color change. Although the as-printed samples were white, the samples heated at 300°C changed color from white to brown (Fig 11), which was an apparent indication of the existence of some organic matters in the commercial starting powder (ZP150) and binder solution (zb63) used in this work.
3. The 1.3% weight loss from 390°C to 520°C could be related to the completion of the removal of the remaining water molecules from the basanite structure and the formation of anhydrite (CaSO_4_).
4. No significant weight change was observed from 520°C to 1000°C because there is no more molecular water to exit or organic binder to burn out, and the anhydrite structure was stable in this temperature range. However, porous samples heat treated at 500 and 900°C could break down easily as shown in Fig 11. Therefore, no compressive strength and elastic modulus measurements could be performed on these samples.

**Fig 12:**
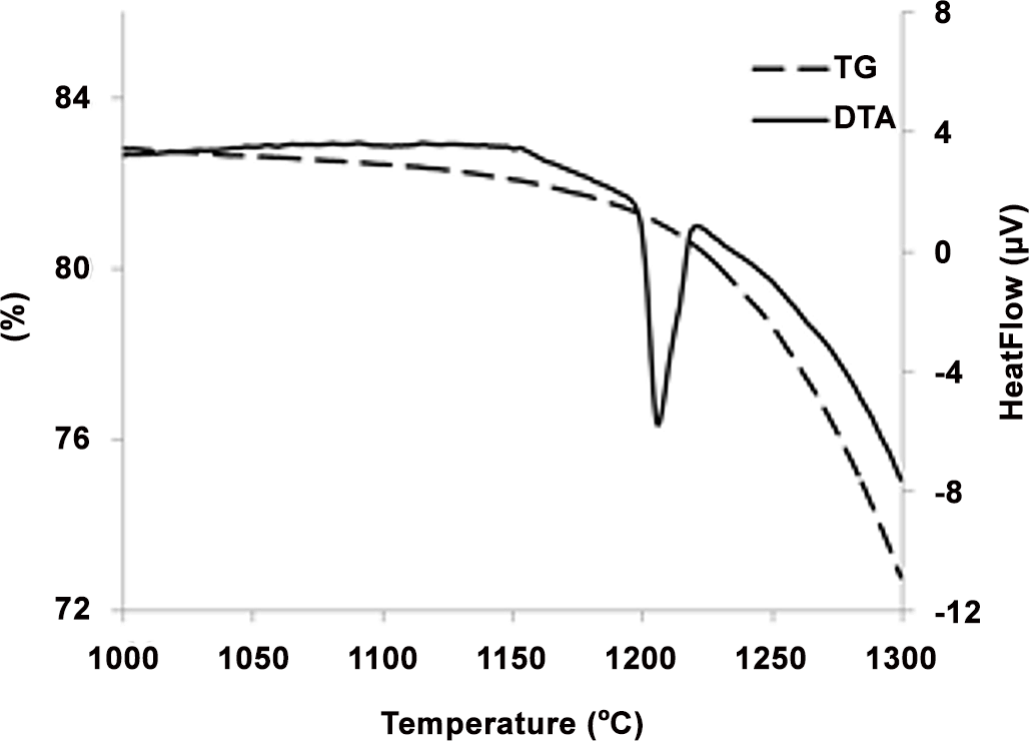
TG-DTA curve of as-printed scaffold.

The thermal events above 1000°C were investigated using a DTA-TG apparatus. Fig 12 shows the DTA-TG curve of the as-printed sample in the temperature range of 1000°C–1300°C. An endothermic peak appeared at 1200°C, which may be attributed to the formation of calcium oxide as a result of the partial thermal decomposition of anhydrous calcium sulfate according to the following reaction [65]:

$${2\text{CaSO}}_{4}\to 2CaO+2SO_{2}+0.5O_{2}$$ (4)

The 1200°C peak was also related to the low- to high-temperature phase transformation of CaSO_4_ at 1200°C, which is reversible during the cooling cycle [66]. The weight loss was more significant after 1200°C, suggesting that the partial decomposition of anhydrite and the formation of CaO were accelerated based on Eq 4. This result was supported by the XRD and SEM-EDS analyses that clearly showed the formation of the CaO phase at 1200°C and above.

**Fig 13:**
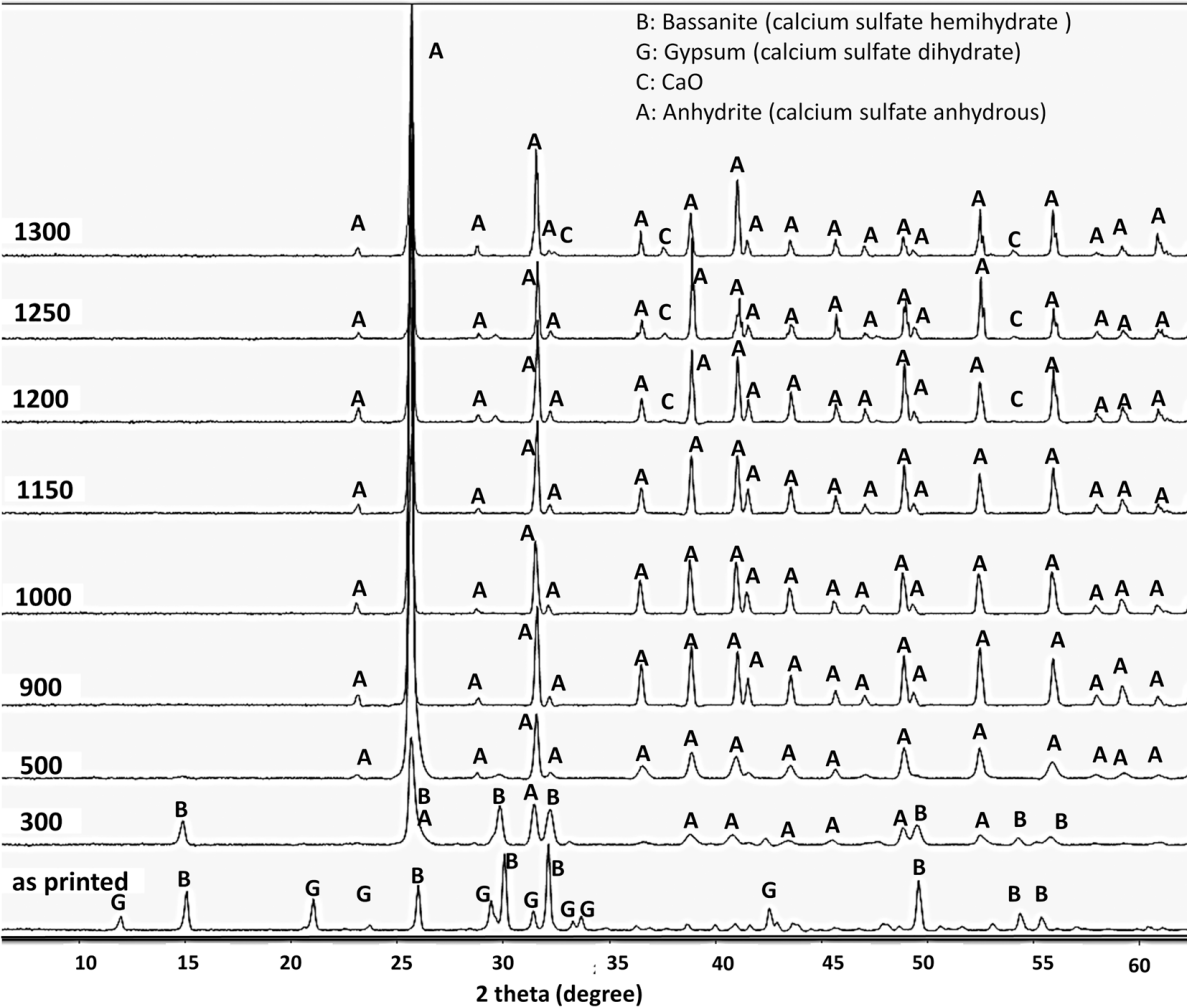
XRD patterns of the printed scaffolds after heat treatment at various temperatures from 300°C to 1300°C.

The XRD patterns of the printed scaffolds after heat treatment at various temperatures from 300°C to 1300°C are shown in Fig 13, with the major peaks labeled. XRD analysis of the as-printed scaffolds prior to heat treatment showed characteristic peaks of dihydrate and hemihydrate CaSO_4_ (designated as G and B in the XRD pattern, respectively). This result disclosed that a part of the zp150 calcium sulfate hemihydrate (CaSO_4_.0.5H_2_O) powder reacted with the water-based binder throughout the 3DP process and formed calcium sulfate dihydrate (CaSO_4_.2H_2_O).

The CaSO_4_.2H_2_O peaks almost disappeared after heat treatment at 300°C. However, the dehydration process was not completed at 300°C, as suggested by traces of some faded peaks of the CaSO_4_.0.5H_2_O phase. All representative peaks of CaSO_4_.2H_2_O and CaSO_4_.0.5H_2_O disappeared when the samples were heated above 300°C, which implied the full removal of water molecules from the chemical structure of the printed calcium sulfate scaffolds.

No clear differences were observed in the XRD patterns of the scaffolds heat-treated at 500°C, 900°C, 1000°C, and 1150°C. The anhydrous form of calcium sulfate (CaSO_4_ or anhydrite) was the only crystalline phase existing in the samples in this temperature range.

**Fig 14:**
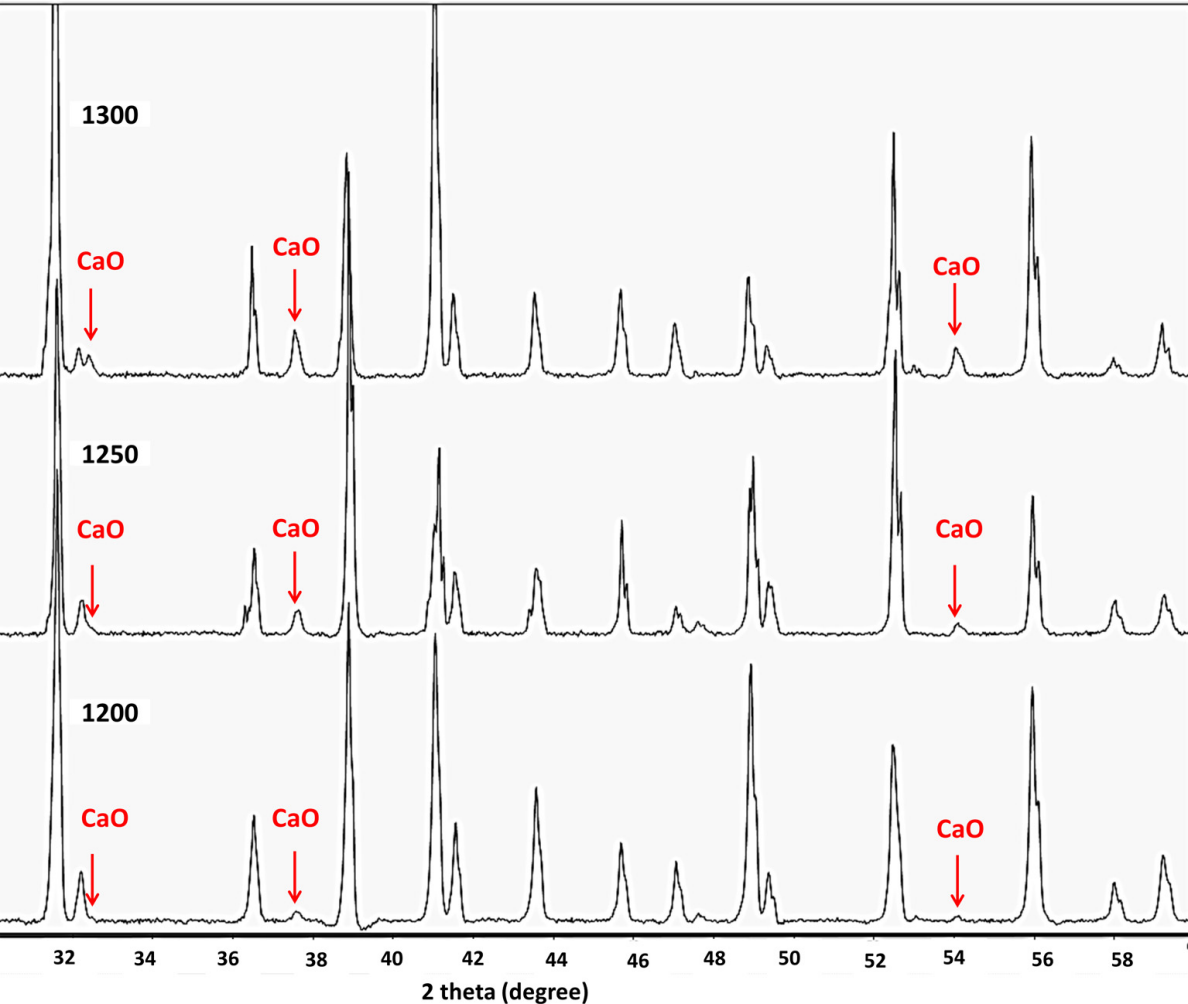
XRD pattern of the printed scaffold after heating at 1200°C, 1250°C, and 1300°C. The appearance of CaO peaks (red arrows) confirms the formation of calcium oxide after the partial thermal decomposition of CaSO_4._

Traces of calcium oxide were observed in the XRD patterns of all scaffolds heat treated at temperatures higher than 1200°C (Fig 14). Correspondingly, SEM micrographs and EDS revealed small scattered CaO regions in the microstructure of the specimens that heat treated at 1200°C, 1250°C, and 1300°C.

The microstructural features and surface topography of the 3DP prototypes and samples heat treated at various temperatures were studied by SEM micrographs obtained in the backscattered mode.

**Fig 15:**
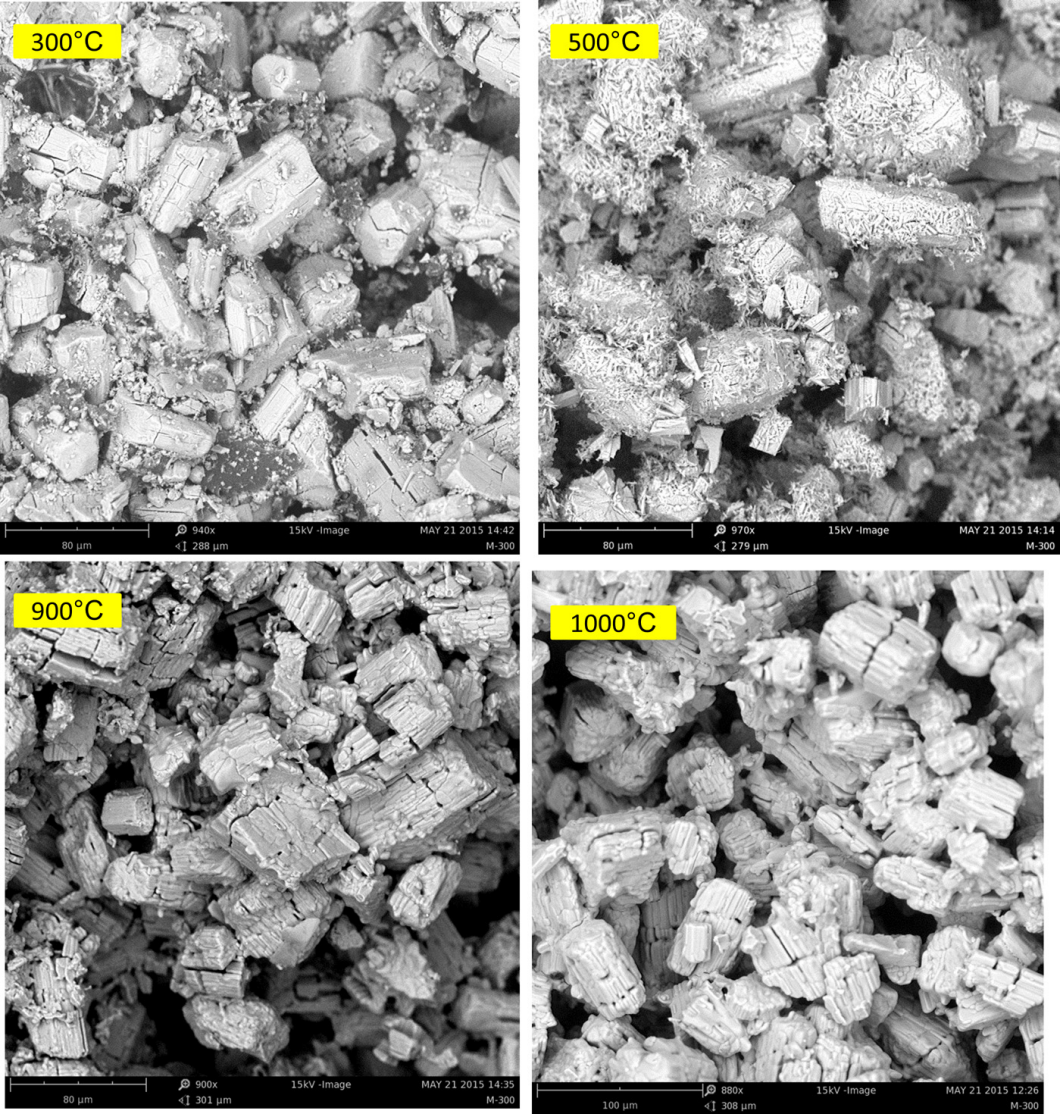
SEM backscattered mode image of the microstructures of the as-printed scaffold and samples heat treated at 300°C, 500°C, 900°C, and 1000°C.

The microstructures of the as-printed scaffold and samples heat treated at 300°C, 500°C, 900°C, and 1000°C are shown in Fig 15.

Anhydrite crystal prisms with relatively regular shape were observed in the microstructure of the sample heated at 300°C. Randomly distributed smaller irregularly formed or broken particles were also observed, which may be attributed to the calcium sulfate hemihydrate phase present in the sample (confirmed by the XRD pattern of the 300°C sample in Fig 13). The hemihydrate phase was almost completely converted to the anhydrous phase when heated to 500°C. As a consequence, the microstructure changed noticeably, and crystals with remarkably different morphologies appeared along with larger particles, similar to those of the 300°C sample. Nevertheless, these particles were not new compositions. The XRD and SEM-EDS results confirmed that the specimen only consisted of anhydrite crystals. The microstructure of the samples heat treated at 900°C and 1000 C looked almost the same. The SEM images showed individual particles that were weakly bonded together. As expected, no sign of densification caused by sintering in the 300°C–1000°C temperature range was observed. The lack of strength was the main feature of the specimens heat treated in this range. Clear evidence of some intergranular cracks in the microstructure of all heat-treated samples was also observed, which may be attributed to the internal stresses induced because of the different calcium sulfate phase transformations that occurred during heat treatment.

**Fig 16:**
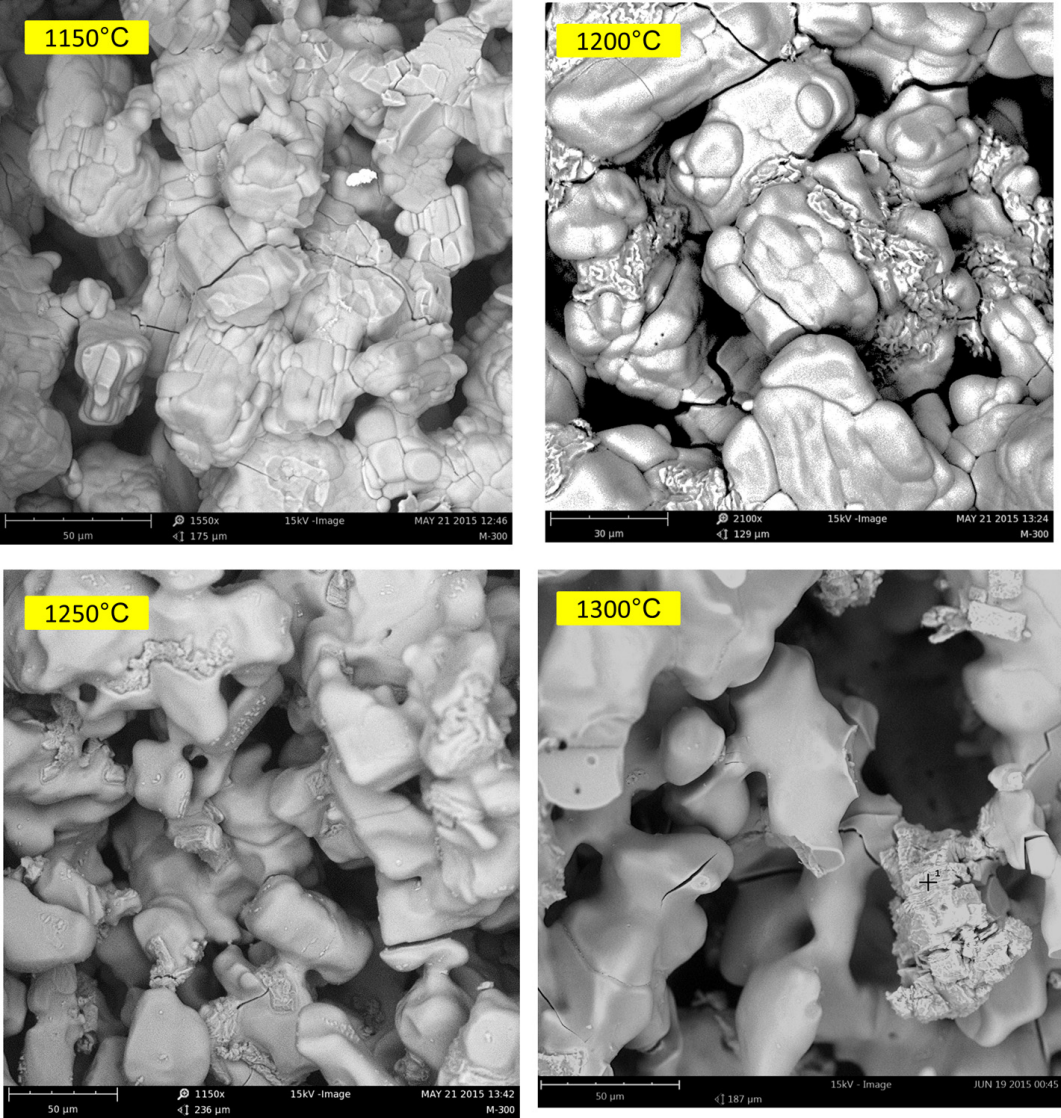
SEM backscattered mode image of the microstructures of the samples heat treated at 1150°C, 1200°C, 1250°C, and 1300°C.

Fig 16 shows the SEM micrographs of the microstructure of the scaffolds heat treated at 1150°C, 1200°C, 1250°C, and 1300°C. Based on the SEM images, although the particles were considerably densified, contact between the particles was insufficient. Therefore, considering the individual particles, the sintering process progressed noticeably. However, the bulk density of the porous samples sintered at 1150°C and above was still far from the density of the solid sample because of their low green bulk density, which was attributed to the presence of induced micro- and predesigned macro-porosity.

The most considerable feature after heating at temperatures higher than 1200°C was the appearance of discrete regions of calcium oxide in the anhydrite microstructure. This finding was in agreement with the information obtained from the XRD patterns of the 1200°C, 1250°C, and 1300°C samples (Fig 14), which was supported by the EDS analysis results.

**Fig 17:**
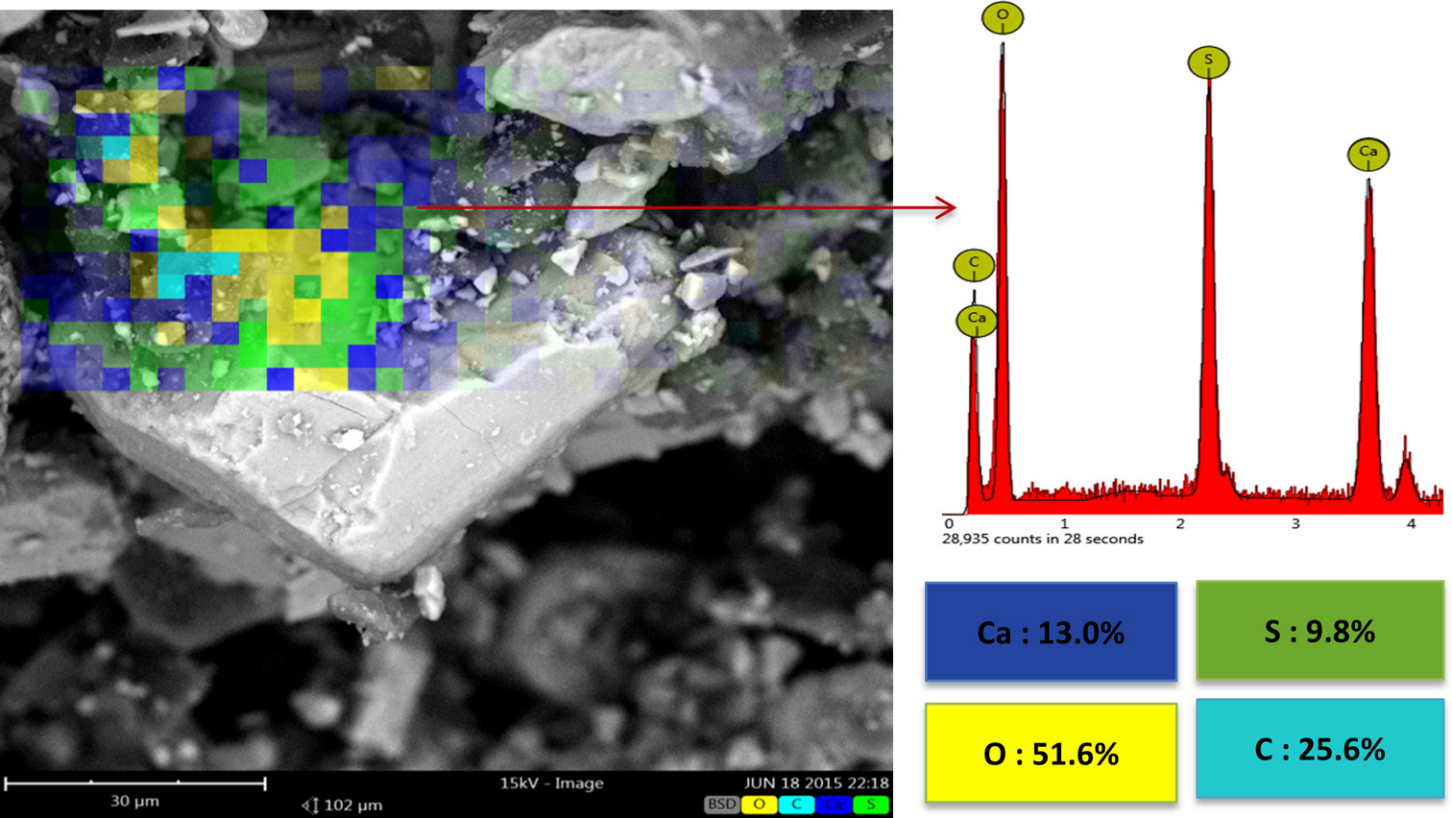
SEM-EDS elemental map analysis of the as-printed scaffold.

SEM-EDS elemental map analysis of the as-printed scaffold is shown in Fig 17. Besides the Ca, S, and O elements that apparently came from the calcium sulfate body, a significant amount of C (25.6%) was also present, which represented the organic constituents of the zp150 powder and the zb63 binder used in this work for 3DP.

**Fig 18:**
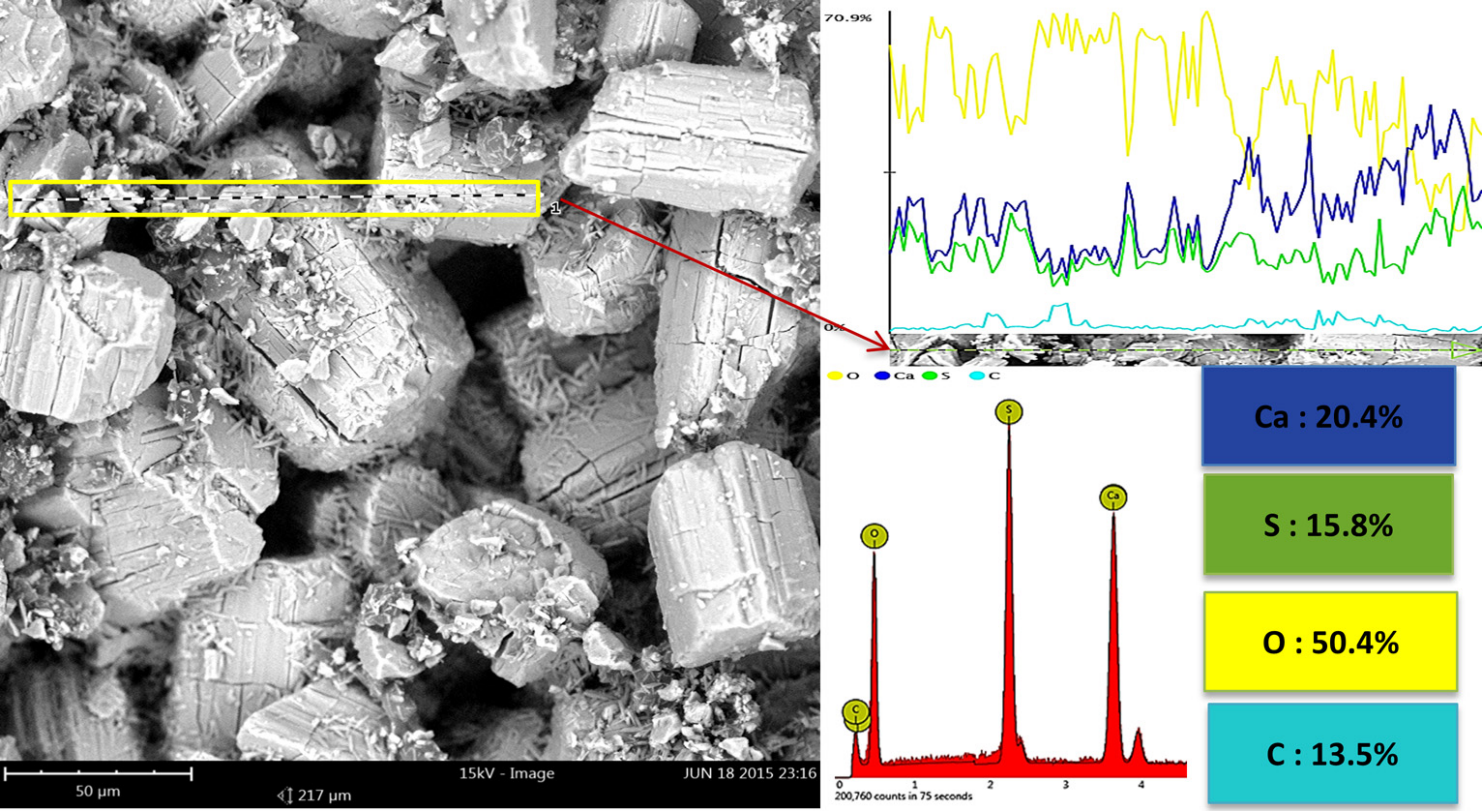
SEM-EDS (line scan) analysis of the specimen heated at 300°C.

SEM-EDS line scan analysis of the sample heated at 300°C is shown in Fig 18. A considerable amount of carbon (13.5%) was detected, which indicated that 300°C was not sufficient to remove all the organic substances present in the printed scaffolds.

**Fig 19:**
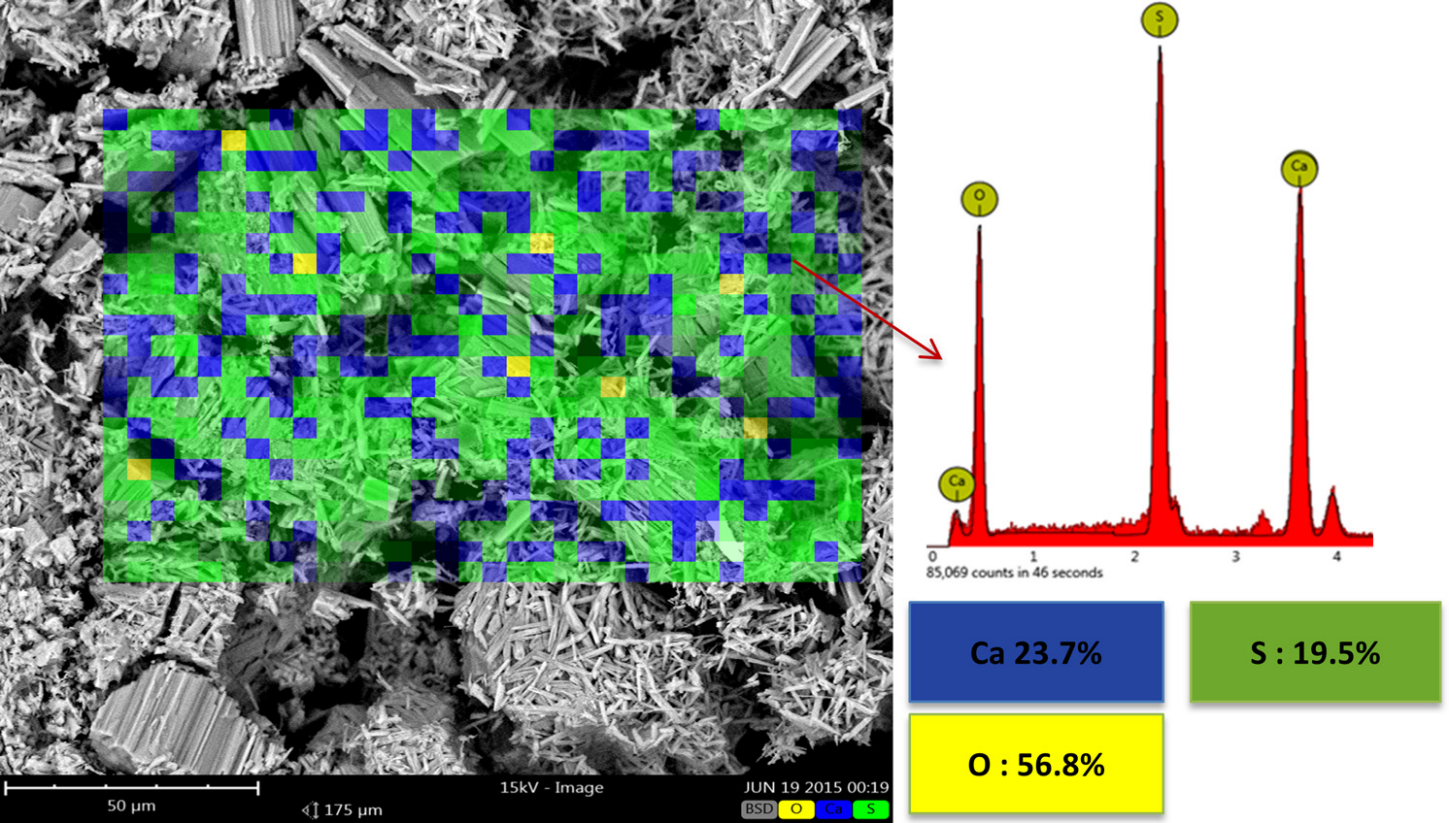
SEM-EDS elemental map analysis of the specimen heated at 500°C.

**Fig 20:**
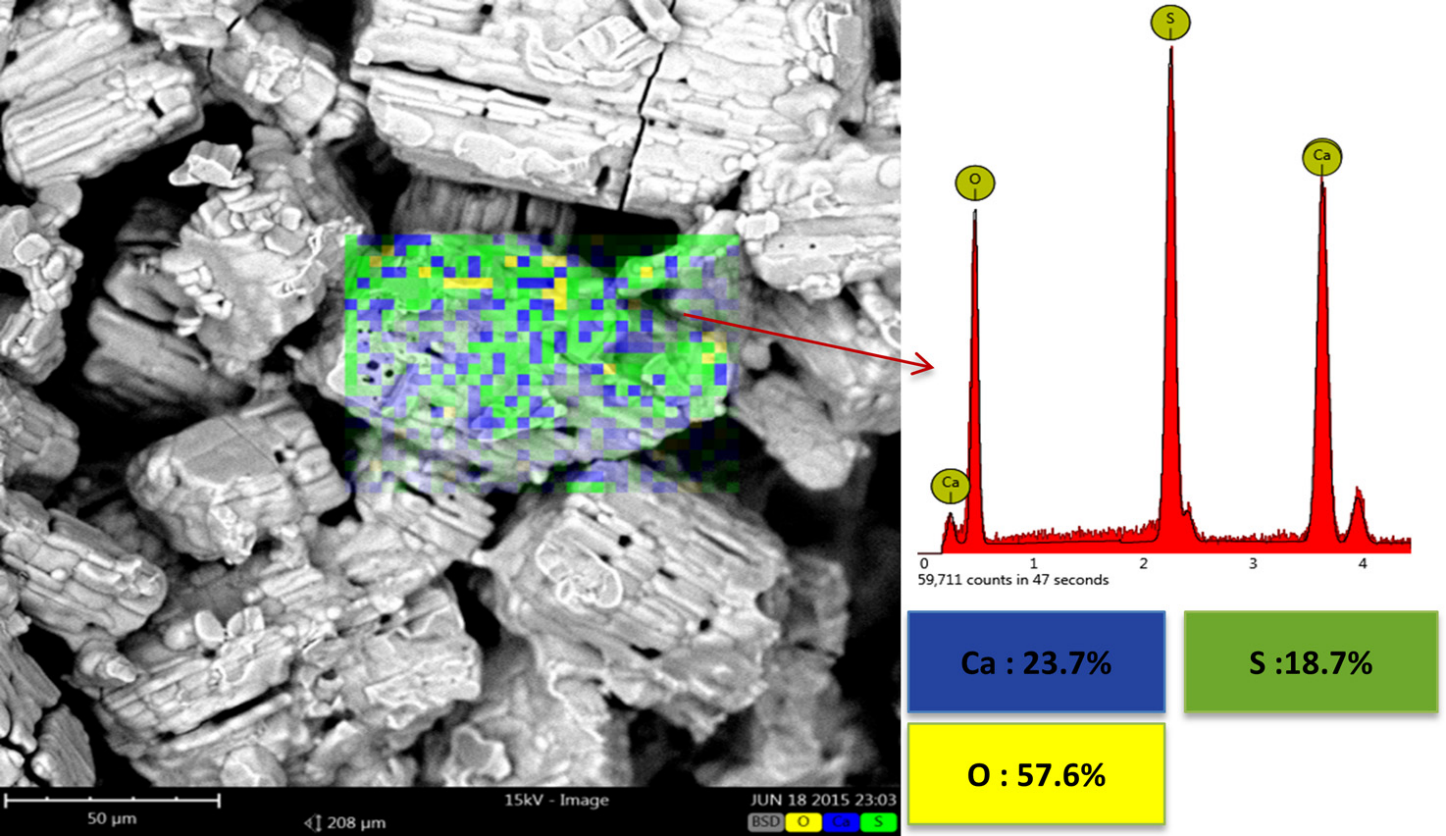
SEM-EDS elemental map analysis of the specimen heated at 1000°C.

Fig 19 shows the result of SEM-EDS elemental map analysis of the sample heat treated at 500°C. Element distribution analysis only showed Ca, S, and O. The total absence of C peaks in the EDS spectra suggested the complete burn out and elimination of organic materials introduced by the starting powder and binder at 500°C. No sign of carbon was observed. The microstructure and SEM-EDS analyses of the samples heat treated at 900°C, 1000°C, and 1150°C were more or less similar to those of the 500°C specimen. The chemical composition of all these samples were almost identical and consisted of anhydrous calcium sulfate (anhydrite) as the only crystalline phase in the structure (see the SEM-EDS elemental map analysis in Fig 20 and XRD patterns in Fig 13).

The printed scaffolds underwent partial decomposition when heated above 1200°C. Calcium oxide appeared in the microstructure of the samples after heat treatment at 1200°C, 1250°C, and 1300°C.

**Fig 21:**
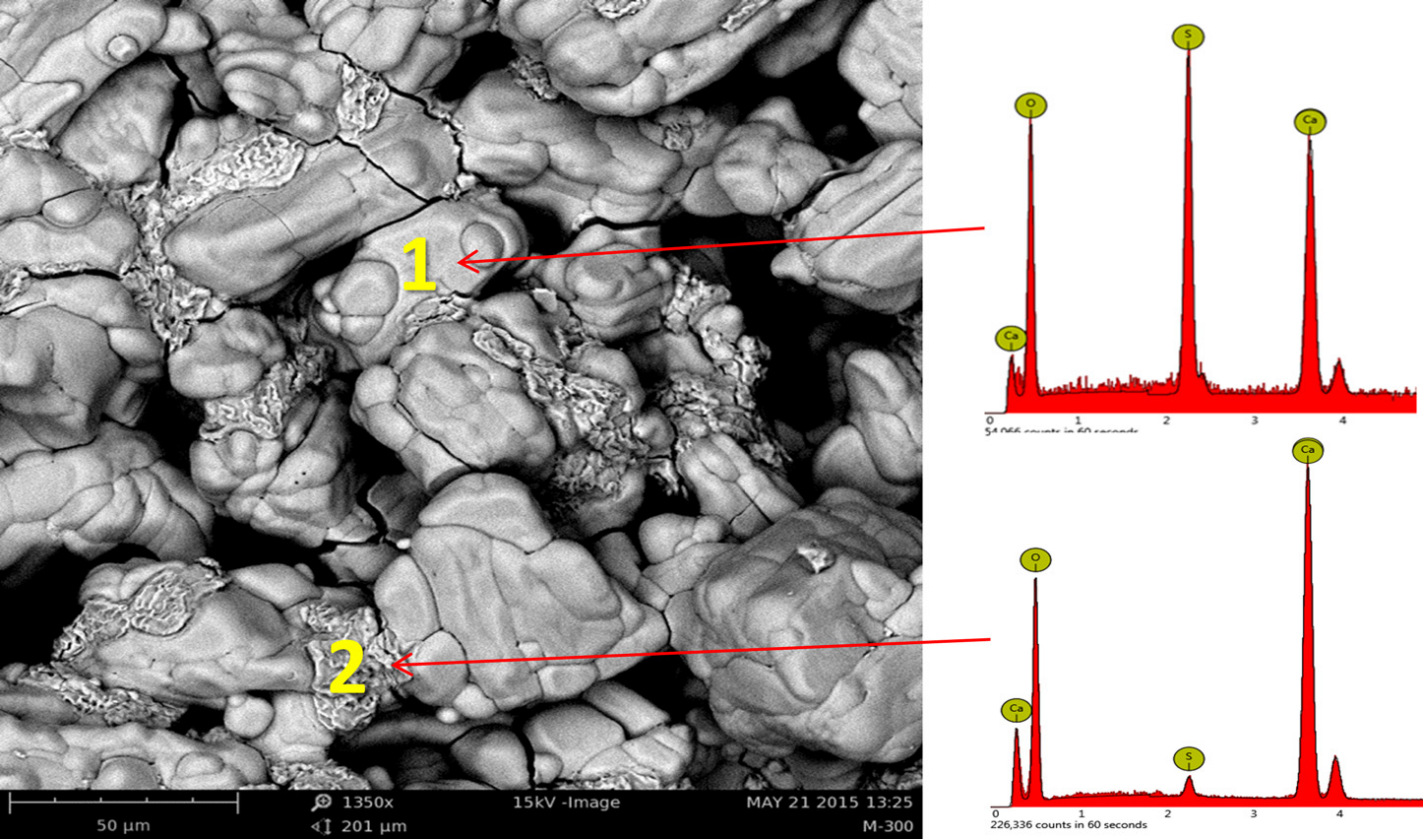
SEM micrograph of the sample heated at 1200°C. Formation of calcium oxide (spot 2) is shown following partial thermal decomposition of CaSO_4_ (spot 1).

The SEM micrograph of the sample heated at 1200°C is shown in Fig 21. Spot EDS chemical analysis confirmed the formation of calcium oxide according to Eq 4. The results of the chemical analysis of two typical CaSO_4_ and CaO spots are shown in Table 2. As anticipated, no significant quantity of sulfur was detected in the spot chemical analysis of the discrete regions attributed to the calcium oxide in the microstructure of the 1200°C sample.

**Table 2:** Chemical analysis of spots 1 and 2 (in Fig 4) attributed to calcium sulfate and calcium oxide in the microstructure of the sample heated at 1200°C.

Element	SPOT 1(%)	SPOT 2(%)
Ca	22.5	34.5
O	60.1	64.2
S	17.4	1.3

### Mechanical features, shrinkage, and density

**Fig 22:**
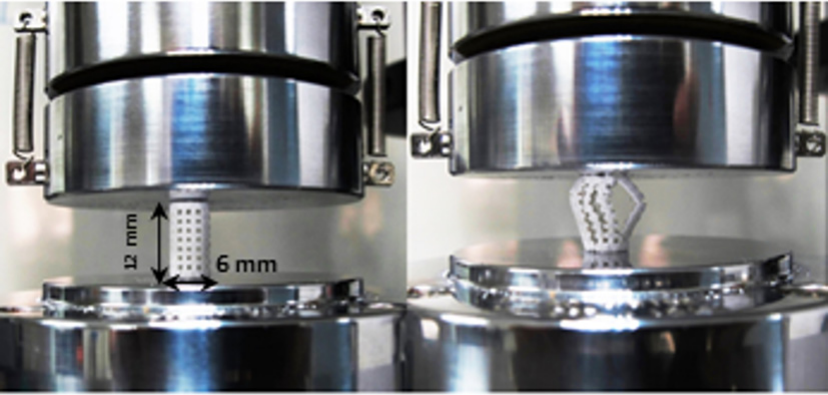
Specimens under compression test.

The mechanical behaviors of the printed and heat-treated scaffolds were investigated using a universal testing instrument with compression test fixtures. The compressive tests were carried out until the sample was broken. Fig 22 shows a typical 3DP test specimen before and after critical failure.

**Fig 23:**
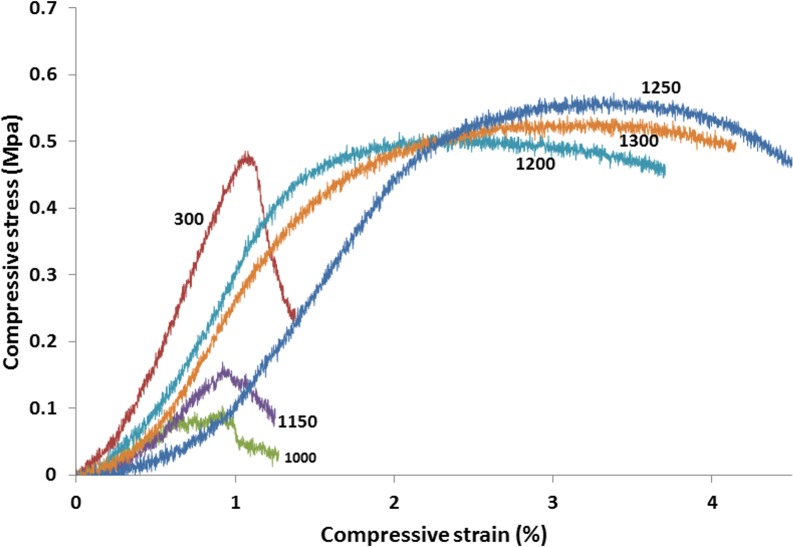
representative Strain–stress curves for porous scaffolds heat treated at 300°C, 1000°C, 1150°C, 1200°C, 1250°C, and 1300°C.

The representative strain–stress curves of the porous scaffolds heat treated at 300°C, 1000°C, 1150°C, 1200°C, 1250°C, and 1300°C are shown in Fig 23. Ultimate compressive strength and compressive elastic modulus were calculated using the maximum compressive stress recorded in the stress–strain curve, as well as the slope of the linear region before the yield point. The scaffolds heat treated at temperatures higher than 1200°C showed an increase in compressive strength and Young’s modulus. An extension of the plastic region was also observed, which suggested higher toughness.

The trend of compressive strength and Young’s modulus change with temperature was similar for solid and porous specimens, although the strength values and the rates of change were lower in the porous samples compared with those in the solid ones.

**Fig 24:**
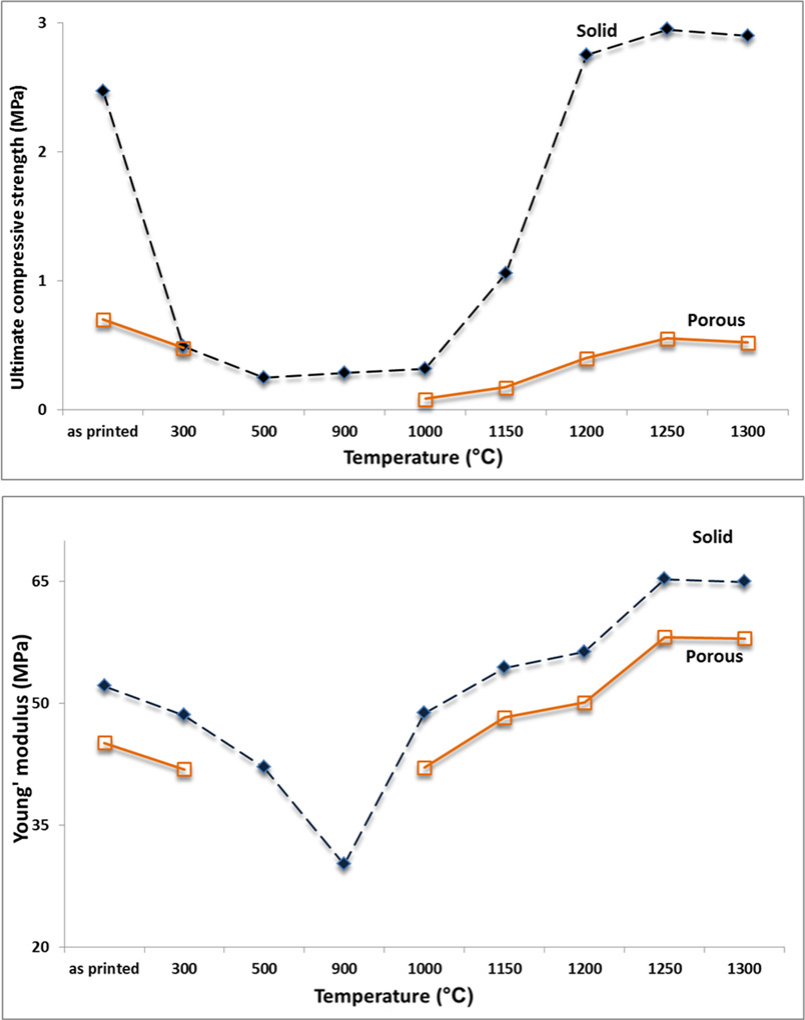
Change in compressive strength and Young’s modulus of the porous and solid specimens with heat treatment temperature.

The results of the compressive strength test, Young’s modulus, and bulk density of the porous and solid samples after heat treatment at various temperatures are summarized in Table 3, and the trends are shown in Fig 24.

**Table 3:** Average value of the compressive strength test, Young’s modulus, and bulk density of the porous and solid samples after heat treatment at various temperatures.

Samples temperature (°C)	Compressive strength(MPa)		Young’s modulus(MPa)		Density (g/cm^3^)	
	Solid	Porous	Solid	Porous	Solid	Porous
**As-printed**	2.47	0.70	52.11	45.13	1.35	0.76
**300**	0.49	0.48	48.56	41.89	1.17	0.646
**500**	0.25	-	42.13	-	1.26	-
**900**	0.29	-	30.25	-	1.31	-
**1000**	0.32	0.08	48.78	42.1	1.33	0.76
**1150**	1.06	0.17	54.36	48.26	1.47	0.786
**1200**	2.75	0.40	56.34	50.1	1.51	0.75
**1250**	2.95	0.55	65.32	58.12	1.22	0.595
**1300**	2.90	0.52	64.98	57.98	1.22	0.59

**Fig 25:**
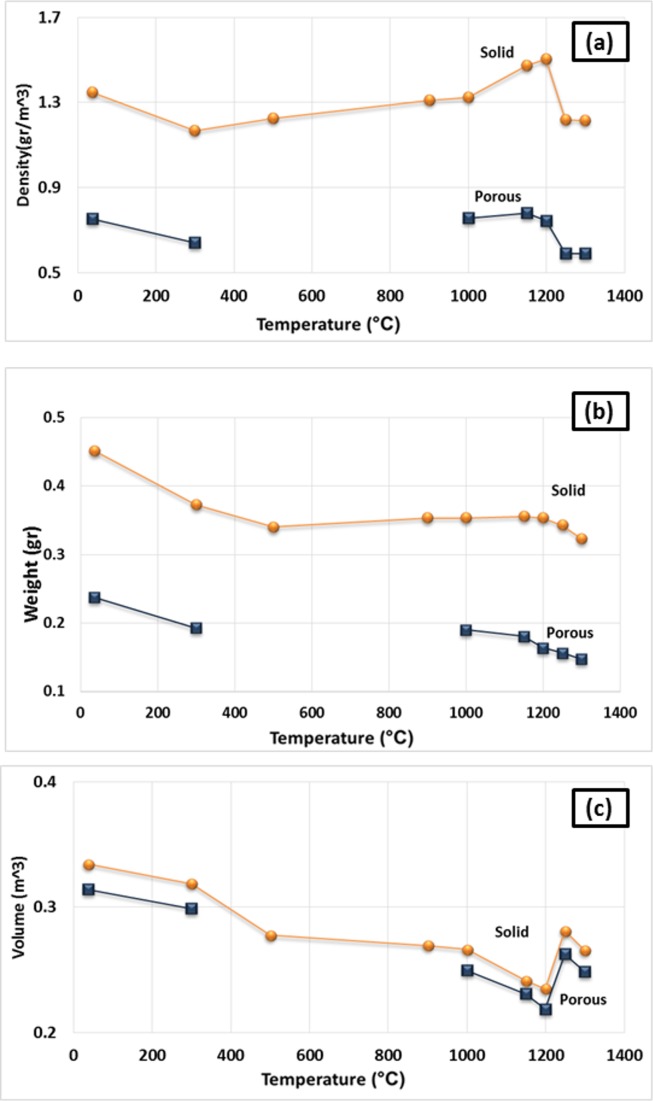
Average value of Bulk density (a), volume (b), and weight (c) of the porous and solid specimens versus heat treatment temperature.

**Fig 26:**
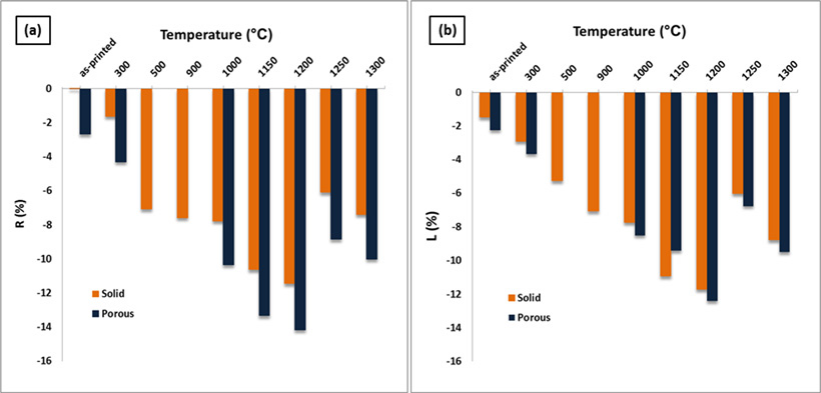
Thickness and diameter shrinkage percentage of the samples as a function of heat treatment temperature.

Shrinkage of the printed porous and solid samples was determined by measuring the diameter and thickness of the cylinder-shaped samples before and after heat treatment compared to the dimension of CAD design. The average values are summarized in Table 4. Fig 25 shows the bulk density, volume, and weight of the porous and solid specimens versus the heat treatment temperature. The thickness and diameter shrinkage percentages of the samples as a function of heat treatment temperature are also shown in Fig 26.

**Table 4:** Shrinkage of the cylinder-shaped porous and solid samples after heat treatment at various temperatures.

Samples-temperature(°C)	R%		L%	
	Solid	Porous	Solid	Porous
**As-printed**	0.017	-2.67	-1.47	-2.25
**300**	-1.62	-4.3	-2.90	-3.67
**500**	-7.05	-9.67	-5.27	-6
**900**	-7.56	-10.17	-7.05	-7.83
**1000**	-7.75	-10.33	-7.72	-8.5
**1150**	-10.62	-13.33	-10.93	-9.42
**1200**	-11.44	-14.17	-11.71	-12.42
**1250**	-6.10	-8.83	-6.02	-6.75
**1300**	-7.37	-10	-8.77	-9.5

Heating the printed scaffolds at 300°C significantly decreased the compressive strength from 2.47 MPa to 0.48 MPa and 0.7 MPa to 0.48 MPa for the solid and porous samples, respectively. The same trend was observed for Young’s modulus. The elastic modulus decreased from 52.11 MPa to 48.56 MPa for the solid samples, and fell from 45.13 MPa to 41.89 MPa for the porous scaffolds. The bulk density of the scaffolds heat treated at 300°C decreased by 18% (from 0.76 g/cm^3^ to 0.64 g/cm^3^) and 12% (from 1.35 g/cm^3^ to 1.17 g/cm^3^) for the solid and porous samples, respectively, compared with the as-printed samples. The significant weight loss caused by decomposition and exit of organic species and dehydration of the starting hemihydrate calcium sulfate were the main reasons for the decrease in density.

Heating the scaffolds at temperatures higher than 300°C resulted in an undesirable mechanical failure. The compressive test could not be performed on the 500°C and 900°C porous samples because they were extremely fragile, weak, and could break down easily (see Fig 11). Therefore, no compressive strength and elastic modulus were documented in this work for the porous samples heat treated at temperatures higher than 300°C and lower than 1000°C. The inadequate mechanical strength of the samples heat treated at this temperature range could be explained by the lack of necessary bonding between the printed layers and adjacent particles. This finding was mainly attributed to the partial combustion of the organic binder at 300°C and its complete removal at 500°C (see the TG-DTG curve in Fig 10 and SEM-EDS elemental analysis in Figs 17, 18 and 19). Below 1000°C, the calcium sulfate system showed no evidence of sintering, and the densification (and increase in strength) of the 3DP prototypes had yet to begin. Thus, practically neither the organic binder nor the sintering process contributed to particle and layer bonding when the printed scaffolds were heat treated at the 300°C–1000°C temperature range.

A very minor increase in the trend of density versus temperature curve was observed from 500°C to 1000°C. No considerable weight loss was observed in this temperature range, and an insignificant density increase was mostly caused by a slight decrease in volume. The XRD and SEM-EDS results showed no phase change, whereas microstructural coarsening began at 900°C. Heating the scaffolds at 1000°C and higher temperatures substantially increased the density and improved the compressive strength and elastic modulus in both porous and solid samples. For the solid samples, the maximum compressive strength (2.95 MPa) and Young’s modulus (65.32 MPa) were achieved at 1250°C, which exceeded the compressive strength (2.47 MPa) and Young’s modulus (52.11 MPa) of the solid as-printed samples. Similarly, the compressive strength of the porous samples increased from 0.08 MPa at 1000°C to 0.55 MPa at 1250°C, which was in a range comparable with that of the initial as-printed scaffold (0.7 MPa). The elastic modulus also increased from 42.1 MPa at 1000°C to 58.12 MPa at 1250°C, which was higher than that of the as-printed scaffold (45.13 MPa). This result was mainly attributed to the progress of the sintering process at temperatures higher than 1000°C, which densified the calcium sulfate scaffolds and improved the mechanical properties of the struts (see microstructures of the scaffolds heat treated at 1150°C, 1200°C, 1250°C, and 1300°C in Fig 16). From 1200°C, a portion of the CaSO_4_ phase decomposed and converted to CaO in expense of the exit of a considerable amount of SO_3_ (Eq 4), resulting in considerable weight loss (Fig 12) and reduction in density. The decomposition reaction is theoretically associated with 58.81% weight loss. From 1250°C to 1300°C, density remained almost unchanged because of a compromise between the acceleration of the sintering process and the weight loss caused by the decomposition of calcium sulfate.

The shrinkage of scaffolds heat treated at 300°C was 4.3% in diameter and 3.67% in height for the porous samples, and 1.62% in diameter and 2.9% in height for the solid samples. The dimensions of the scaffolds remained relatively constant between 300°C and 900°C. From 900°C to 1200°C, the diameter and height of the porous samples experienced 14.17% and 12.42% shrinkage, respectively. From 1200°C to 1250°C, although the progress of sintering at high temperatures was expected to increase shrinkage, the samples practically shrunk less. A trade-off was observed between the acceleration of the sintering process and the weight loss and volume decrease caused by the decomposition of calcium sulfate that resulted in decreased shrinkage. Further heating from 1250°C to 1300°C facilitated the sintering process and increase in shrinkage.

To find the significance level, ANOVA was performed for dimension and weight tests. The results are summarized in Table 5. The SS, df, and F correspond to the sum of squares, degree of freedom, and F value, respectively. The ANOVA results demonstrated that the tests were highly significant, with P < 0.001.

**Table 5:** Results of ANOVA for diameter, height, and weight.

	Diameter				Height				Weight			
Source of variation	SS	df	Variance	F	SS	df	Variance	F	SS	df	Variance	F
**Between group**	4.01	8	0.50	140.57	13.19	8	1.64	525.65	0.11	8	0.01	414.00
**Within group**	0.29	81	0.00	-	0.25	81	0.00	-	0.00	81	0.00	-
**Total**	4.29	89	-	-	13.44	89	-	-	0.11	89	-	-

### *In vitro* evaluation of the as-printed and heat-treated scaffolds

**Fig 27:**
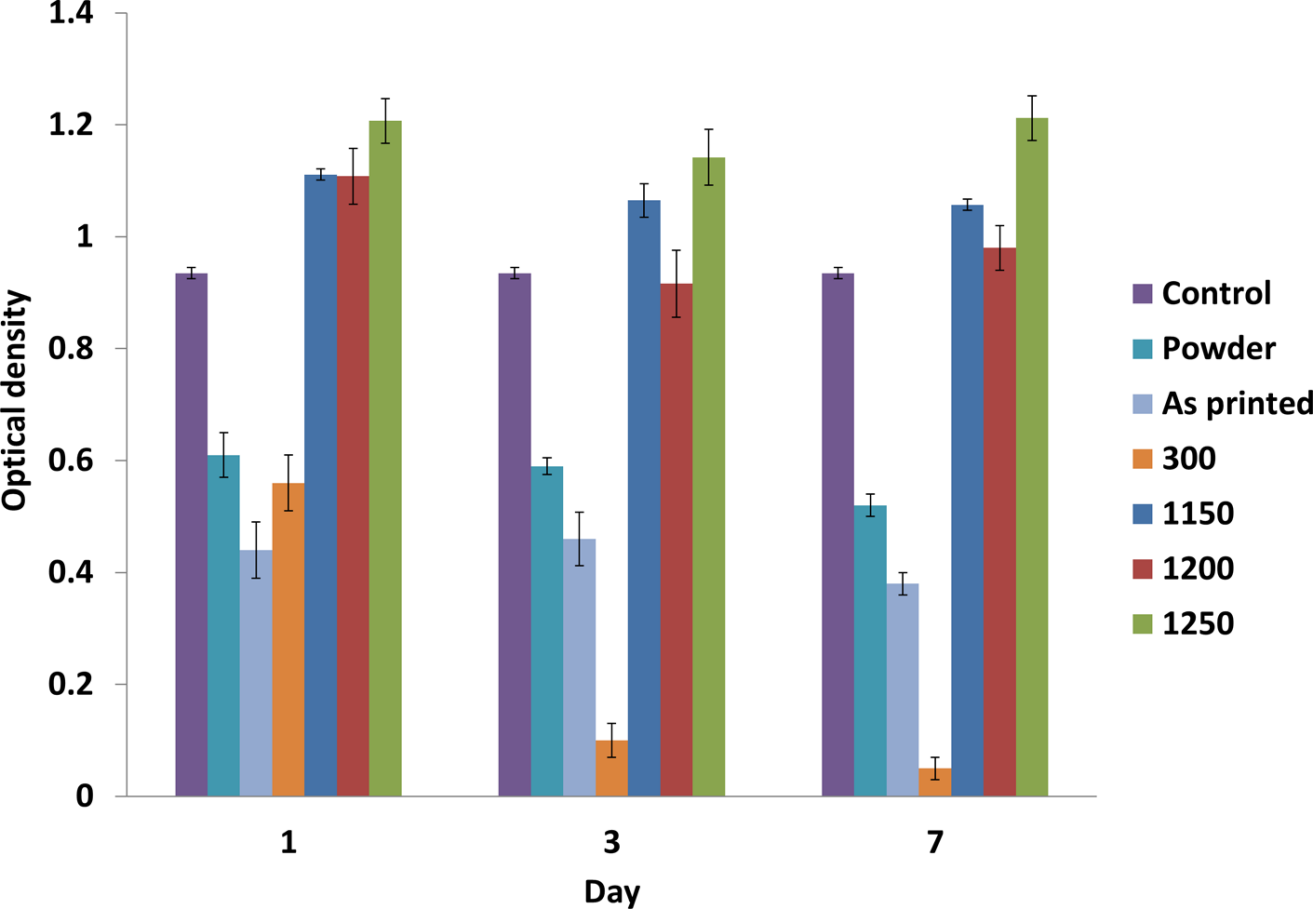
Results of MTT assay on the ZP150 powder, as-printed scaffold, and samples heated at 300°C, 1150°C, 1200°C, and 1300°C.

The MTT measurement results are shown in Fig 27. According to the standard protocols, the extracts of the powders were collected on days 1, 3, and 7. Subsequently, MG63 cells were exposed to different extractions of samples for 24 h. The results showed a reduction in viability for the powder and as-printed samples below 60%. This reduction was attributed mainly to the composition of the as-printed scaffolds (Fig 17) because of the presence of organic constituents in the zp150 powder and zb63 binder used in this work for 3DP.

The sample heat treated at 300°C had significant toxic effects on cells, which was mainly caused by the products resulting from the partial decomposition and combustion of the organic materials present at the commercial binder (zb63) and powder (zp150). Hence, this temperature may not be sufficient for the complete combustion of organic additives.

To completely eliminate the organic additives and improve the mechanical properties of the samples, the as-printed samples were heat treated at a higher temperature. The samples heat treated at 1150°C, 1200°C, and 1250°C showed significantly greater viability compared with the control, as shown in Fig 27. This result may be due to the complete removal of the toxic organic species during the high-temperature heating process.

## Conclusion

3D-printed calcium sulfate scaffolds are promising biomaterial in BTE. However, they still require much improvement in properties in terms of BTE application requirements. A post-processing approach is usually employed to improve the physical, chemical, and biological properties of 3DP scaffolds. In this study, we investigated the effect of heat treatment on the structural, mechanical, and physical properties of calcium sulfate prototypes fabricated using 3DP.

The results of different microscopy, spectroscopy, and biological characterization techniques showed that the as-printed scaffolds and specimens heat treated at 300°C exhibited severe cytotoxicity in vitro but possessed almost adequate compressive strength. Heat treatment of the specimens at the 500°C, 900°C and 1000°C temperature range resulted in less cytotoxic scaffolds with insufficient mechanical strength. This result was attributed to the lack of binding strength between the particles and layers because of the exit of the organic binder before 500°C and insufficient densification below 1000°C. With progress of the sintering process at temperatures higher than 1000°C, higher compressive strength and greater viability were achieved. The as-printed sample was mainly composed of the hemihydrate calcium sulfate phase. Anhydrous calcium sulfate was the only crystalline phase existing in the samples heated at 500°C, 900°C, 1000°C and 1150°C. At temperatures higher than 1200°C, a part of the calcium sulfate decomposed to calcium oxide and sulfur oxide, resulting in considerable weight loss.

A substantial improvement in viability of the heat-treated scaffolds was observed in this study, even though the compression strength was not improved significantly compared with that of natural bone. Limitations in mechanical properties are still present in this study, which call for future studies. Nevertheless, we consider that the findings of this study give a better insight into the complex nature of the process of fabrication of synthetic bone grafts and scaffolds via post treatment of 3DP calcium sulfate prototypes.
